# CDK4/6 inhibitors synergize with radiotherapy to prime the tumor microenvironment and enhance the antitumor effect of anti-PD-L1 immunotherapy in triple-negative breast cancer

**DOI:** 10.1186/s12929-025-01173-3

**Published:** 2025-08-20

**Authors:** Wen-Chi Yang, Ming-Feng Wei, Ying-Chun Shen, Chiun-Sheng Huang, Sung-Hsin Kuo

**Affiliations:** 1 Division of Radiation Oncology, Department of Oncology, National Taiwan University Hospital, No. 7, Chung-Shan South Road, Taipei, 100 Taiwan; 2 Graduate Institute of Oncology, National Taiwan University College of Medicine, Taipei, Taiwan; 3 Department of Radiation Oncology, National Taiwan University Cancer Center, National Taiwan University Hospital and National Taiwan University College of Medicine, Taipei, Taiwan; 4 Department of Medical Oncology, National Taiwan University Cancer Center, National Taiwan University Hospital and National Taiwan University College of Medicine, Taipei, Taiwan; 5 Department of Surgery, National Taiwan University Hospital and National Taiwan University College of Medicine, Taipei, Taiwan; 6 Cancer Research Center, National Taiwan University College of Medicine, Taipei, Taiwan; 7 NTU Radiation Application and Hardness Technology Research Center, National Taiwan University, Taipei, Taiwan

**Keywords:** CDK4/6 inhibitor, Radiotherapy, Immunotherapy

## Abstract

**Background:**

Triple-negative breast cancer (TNBC) has the highest mortality rate among all breast cancer subtypes. Although immunotherapy shows promise, its efficacy varies. CDK4/6 inhibitors can radiosensitize and modulate the immune system, and high-dose radiotherapy (RT) can enhance the effects of immunotherapy. This study explored the combination of RT with CDK4/6 inhibitors to improve TNBC immunotherapy by modulating the tumor microenvironment.

**Methods:**

We assessed the radiosensitizing effects of abemaciclib (a CDK4/6 inhibitor) using clonogenic assays in three human TNBC cell lines (MDA-MB-231, MDA-MB-453, and MDA-MB-468) and two murine TNBC cell lines (4T1 and EMT6). The antitumor efficacy of the treatments (control, RT, abemaciclib, anti-PD-L1 antibody [aPD-L1], abemaciclib combined with aPD-L1, abemaciclib combined with RT, aPD-L1 combined with RT, and the triple combination of abemaciclib with aPD-L1 and RT) was evaluated in both 4T1 and EMT6 cell line-derived immunocompetent mouse models. Interferon-γ (IFN-γ) levels in mouse blood were monitored to gauge the immune response. Tumor-infiltrating lymphocytes (TILs) were analyzed using flow cytometry and immunohistochemical staining.

**Results:**

Clonogenic assays showed synergistic effects of RT and abemaciclib in all TNBC cell lines. RT increased PD-L1 expression, whereas abemaciclib did not alter PD-L1 expression. In the 4T1 and EMT6 mouse models, the triple combination treatment markedly inhibited tumor growth (*P* < 0.01). In the 4T1 mouse model, the triple combination group exhibited significantly greater circulating IFN-γ levels (*P* < 0.001) than the other groups. TIL analysis revealed a significant increase in CD4 + and CD8 + T cells and tumor-associated macrophages (*P* < 0.01) in the triple combination therapy group. Immunohistochemical staining confirmed increased infiltration of CD4 + T cells, CD8 + T cells, monocyte chemoattractant protein-1, CD80-, and iNOS- positive macrophages into the tumor microenvironment of this group, with a marked reduction in CD206-positive macrophages.

**Conclusion:**

Combining CDK4/6 inhibitors with RT enhanced the antitumor effects of aPD-L1 immunotherapy against TNBC. This effect was correlated with increased IFN-γ secretion and recruitment of CD4 + and CD8 + T cells and M1 tumor-associated macrophages, leading to modulation of the tumor microenvironment.

**Supplementary Information:**

The online version contains supplementary material available at 10.1186/s12929-025-01173-3.

## Background

Breast cancer represents the most common cancer among women globally, classified into subtypes based on molecular characteristics that remarkably influence prognosis [1]. Among these, triple-negative breast cancer (TNBC) stands out due to its aggressive nature and a lack of well-defined molecular targets, contributing to poorer outcomes than those in the other three subtypes [2, 3].

The advent of immunotherapy has revolutionized oncological treatment, with immune checkpoint inhibitors (ICIs) emerging as promising treatment options. However, recent studies have demonstrated that the distinct genetic subtypes of TNBCs have a unique tumor microenvironment, impacting the effectiveness of immune responses to treatment [4]. Single-agent anti-PD-L1 therapies, such as atezolizumab, have demonstrated limited efficacy in patients with TNBC [5]. Combining ICIs with other anti-cancer treatments has yielded more potent responses, possibly due to increased antigenicity and adjuvanticity [6]. Notably, neoadjuvant chemotherapy combined with pembrolizumab has achieved higher rates of pathological complete response in early TNBC than chemotherapy alone [7], highlighting the need for combination therapies in enhancing immune response.

Cyclin D-cyclin-dependent kinase 4 and 6 (CDK4/6) are pivotal regulators of cell cycle progression, explicitly facilitating transition from the G1 to S phase. Given their crucial role in cell proliferation, CDK4/6 s have emerged as promising therapeutic targets in cancer treatment [8]. In breast cancer, the CDK4/6-cyclin D axis plays a significant role in tumorigenesis [9, 10], with cyclin D1 frequently overexpressed, particularly in estrogen receptor (ER)-positive cases [11].

A previous preclinical study demonstrated that CDK4/6 inhibitors could inhibit tumors in non-luminal cell lines [12]. Despite frequent retinoblastoma (RB) loss that can make patients with TNBC poor candidates for receiving CDK4/6 inhibitors, some studies have shown that TNBC cell lines are sensitive to CDK inhibition in vitro and in vivo [13, 14]. A randomized phase II trial by Tan et al*.* demonstrated that patients receiving trilaciclib, a CDK4/6 inhibitor, alongside chemotherapy achieved better progression-free and overall survival compared to those receiving chemotherapy alone [15], suggesting the potential application of CDK4/6 inhibitors in TNBC treatment.

CDK4/6 inhibitors promote cytotoxic T-cell activity and increase tumor antigen presentation, potentially improving immunotherapy efficacy [16]. Clinical trials, such as the neoMONARCH phase II neoadjuvant study, have also demonstrated upregulation of inflammatory and T-cell-related pathways following treatment with abemaciclib and anastrozole, supporting their potential immune regulation effects in the clinical practice [17].

Radiotherapy (RT) is a common treatment strategy for solid cancers, including breast cancer [18]. High-dose RT exhibits immunogenic properties, acting as an in situ tumor vaccine by stimulating the release of tumor-associated antigen, recruiting cytotoxic T lymphocytes, and enhancing the abscopal effect [19]. Ho et al. conducted an early phase II trial that combined pembrolizumab with hypofractionated RT in patients with metastatic TNBC [20]. The overall response rate was 17.6% in the entire cohort, with three patients achieving complete response outside the radiation field [20]. Early preclinical models have reported synergistic effects between RT and CDK4/6 inhibitors in various cancers, suggesting that combining these treatments could enhance cytotoxicity and potentially modulate the immune response [21, 22].

To identify the best synergistic treatment combination for TNBC, we investigated the effectiveness of combining CDK4/6 inhibitors, RT, and ICIs. TNBC is an aggressive and heterogeneous cancer with limited response to single-agent ICIs such as pembrolizumab and atezolizumab [5, 23]. However, combining ICIs with chemotherapy has resulted in improved outcomes, underscoring the need for exploring combination therapies. CDK4/6 inhibitors not only induce cell cycle arrest but also enhance immune responses by promoting cytotoxic T-cell activity. Similarly, RT boosts immunogenicity by stimulating the release of tumor-associated antigens and recruiting cytotoxic T lymphocytes [19]. Therefore, the combination of a CDK4/6 inhibitor, RT, and ICIs represents a promising approach for TNBC treatment. In the present study, we used a poorly immunogenic and highly metastatic 4T1 mouse model [24, 25] and a relatively immunogenic EMT6 mouse model [26] to mimic advanced human TNBC and thereby investigate whether combining CDK4/6 inhibitors with RT can improve the efficacy of anti-PD-L1 immunotherapy in 4T1- and EMT6- derived immunocompetent mouse models.

## Methods

### Breast cancer cell lines and cell culture

We used three human TNBC cell lines and two murine TNBC cell lines from the American Type Culture Collection (ATCC) (Rockville, Maryland, USA). These human TNBC cell lines, included MDA-MB-231 (ATCC® HTB-26™), MDA-MB-453 (ATCC® HTB-131™), and MDA-MB-468 (ATCC® HTB-132™). We also used two murine TNBC cell lines, including 4T1 (ATCC® CRL-2539™) and EMT6 (ATCC® CRL-2755™). All TNBC cell lines were cultured at 37 °C with 5% atmospheric CO_2_ following the manufacturer’s guidelines for maintenance. The MDA-MB-453, MDA-MB-468, and 4T1 cell lines were cultured in Gibco Roswell Park Memorial Institute 1640 medium, while the MDA-MB-231 cell line was grown in Dulbecco’s modified Eagle’s medium, and the EMT6 cell line was grown in Waymouth’s MB 752/1 medium.

### Drugs and ionizing irradiation

This study investigated the effects of abemaciclib, a CDK4/6 inhibitor, on cellular functions and its potential antitumor activity. Abemaciclib was purchased from a commercial supplier, MedChemExpress (MCE*,* Shanghai, China), prepared as a stock solution in 100% DMSO at a concentration of 10 mmol/L. Ionizing radiation treatment was delivered using a Caesium-137 (Cs137) irradiator (IBL 637, CIS Bio International, Saclay, France) [27] at a dose rate around 3 Gy/min to whole body with 5 mm lead shielding applied to protect the non-target areas. Additionally, the efficacy of immunotherapy was evaluated in vivo using an anti-mouse PD-L1 antibody (catalog number BE0101, BioCell).

Abemaciclib was applied in vitro at 100–200 nM—approximately ≤ 10% of the half-maximal inhibitory concentration (IC_50_), for inhibiting proliferation of our TNBC cell lines (Supplementary Fig. 1)—to permit radiosensitization read-outs without confounding effects from direct cytotoxicity [22]. TNBC cells were irradiated with a single dose of 8 Gy or 16 Gy, administered 1 h after drug treatment. These high single fraction RT doses are widely used to induce DNA damage, upregulate PD-L1 expression, and modulate the immune response [22].

### Cell proliferation assay

The five TNBC cell lines (MDA-MB-231, MDA-MB-453, MDA-MB-468, 4T1, and EMT6) were seeded in 96-well plates at 1000 cells per well in 100 μL medium. Subsequently, the cells were treated with varying concentrations of abemaciclib. In the present study, we assessed cell proliferation 3 days after treatment using the CellTiter 96® Aqueous Non-Radioactive Cell Proliferation Assay (Promega, Madison, Wisconsin, USA). Absorbance readings were obtained at 450 nm using a multi-well plate reader, with a background control for comparison. The IC_50_ of abemaciclib for each cell line was determined based on these measurements.

### Clonogenic assay

The TNBC cell lines MDA-MB-231, MDA-MB-453, MDA-MB-468, 4T1, and EMT6 were cultured in individual wells of six-well plates, with each well containing 2,000 cells. These cells were subjected to various doses of RT, ranging from 0 to 6 Gy, and treated with different concentrations of abemaciclib 1 h prior to RT. The culture medium was maintained until the end of the study. After 7–14 days, the colonies in each well were counted using an inverted phase-contrast microscope at a magnification of 100 ×. A colony was defined as a cluster of more than 50 cells. To assess the synergistic effect of the combined treatments, the combination index (CI) was determined using the Chou–Talalay equation [28].

### Flow cytometry

To assess PD-L1 surface expression, MDA-MB-231, MDA-MB-453, MDA-MB-468, 4T1, and EMT6 cells were exposed to various doses of RT and abemaciclib and harvested after 24 h. The cells were then stained with an anti-PD-L1 antibody (aPD-L1) (#13684; Cell Signaling Technology) for analysis using an LSR II flow cytometer (BD Biosciences, San Diego, CA, USA). To examine tumor-infiltrating lymphocytes (TILs), single-cell suspensions were prepared from tumors dissected from the 4T1 cell line-derived xenograft immunocompetent mice. These cells were stained with fluorochrome-conjugated antibodies targeting CD45 (cat. 536,709, BD Biosciences), CD4 (cat. 553051, BD Biosciences), CD8 (cat. 552877, BD Biosciences), CD11b (cat. 564985, BD Biosciences), GR-1 (cat. 552093, BD Biosciences), F4/80 (cat. 565613, BD Biosciences), Foxp3 (cat. 12-5774-83, eBiosciences), Ly6C (cat. 562737, BD Horizon™), Ly6G (cat. 563011, BD Horizon™), and isotype control RatIgG2a (cat. 12-4321-80, eBiosciences). We distinguished live cells from dead using the LIVE/DEAD™ Fixable Green Dead Cell Stain Kit (cat. L23101, Invitrogen, Waltham, MA). For intracellular markers, cells were fixed and permeabilized with specific solutions (eBiosciences, San Diego, CA, USA) at room temperature for 30 min (extended to 60 min for Foxp3 staining), with a gating set using matched isotype controls. The LSR II flow cytometer (BD Biosciences, San Diego, CA, USA) facilitated the analysis, and all flow cytometry data were processed using FlowJo 10.1 software (Ashland, OR, USA).

### Western blotting

Protein samples from MDA-MB-231, MDA-MB-453, MDA-MB-468, 4T1 and EMT6 cells were harvested following treatment with the control, RT, abemaciclib, or a combination of abemaciclib and RT. Mammalian protein extraction reagent (M-PER; Pierce, Rockford, IL, USA) was used for protein extraction. Equal amounts of protein extracted from each cell line were separated via electrophoresis on 10% sodium dodecyl sulfate–polyacrylamide gel electrophoresis gels using the Tris–glycine buffer system. The separated proteins were transferred onto nitrocellulose membranes (Novex, San Diego, CA, USA) and blocked overnight in 1 × Tris-buffered saline supplemented with 0.1% Tween-20 and 5% nonfat dry milk. The membranes were then probed with primary antibodies against RB (#9309S, Cell Signaling Technology), phosphorylated RB (#8516S, Cell Signaling Technology), and α-tubulin (#2144, Cell Signaling Technology). Following incubation with secondary antibodies, the protein bands were visualized using an enhanced chemiluminescence detection system (Boehringer Mannheim, Germany). Each experiment was conducted at least thrice to ensure reliability.

### Enzyme-linked immunosorbent assay (ELISA)

Serum samples from 4T1 mouse model were analyzed for interferon-gamma (IFN-γ) levels using the Quantikine™ ELISA Mouse IFN-γ Immunoassay kit (cat. MIF00-1; Sigma-Aldrich) following the manufacturer’s protocol for reagent preparation. The assay procedure involved the sequential addition of assay diluent, standards, controls, or samples to the wells, followed by a 2 h incubation at room temperature. After a thorough washing step, mouse IFN-γ conjugates were added, and the plates were incubated for an additional 2 h. Subsequently, a substrate solution was added, and the plates were shielded from light. The optical density at 450 nm of each well was measured using a microplate reader, and this optical density was used to calculate the concentration of IFN-γ present in the samples.

### In vivo mice model

Female BALB/c mice aged 5–6 weeks were obtained from the National Laboratory Animal Center, Taiwan, and the experimental procedures were approved by the Institutional Animal Care and Use Committee of the National Taiwan University (IACUC Number 20210366). Each mouse received a 100 µL subcutaneous injection of prepared 4T1 or EMT6 cells at a concentration of 1 × 10^5^ cells/mL at flank. Tumor volumes were calculated using the following formula: π/6 × length × width × height, with “length,” “width,” and “height” representing the tumor’s respective dimensions. Upon reaching an average tumor volume of approximately 100 mm^3^, the mice were divided into eight treatment groups: control (PBS for anti-PD-L1, DMSO for Abemaciclib), RT alone, abemaciclib alone, aPD-L1 alone, abemaciclib combined with aPD-L1, abemaciclib combined with RT, aPD-L1 combined with RT, and abemaciclib combined with aPD-L1 and RT.

Tumor irradiation was performed using a Cs137 irradiator, delivering 8 Gy weekly for two weeks to maximize immunogenic cell death and limit normal tissue injury; hypofractionated RT schedules of approximately 8–10 Gy per fraction have been shown to potentiate systemic immune responses and synergize with immune checkpoint blockade [29, 30]. Abemaciclib was administered orally daily for two weeks at 50 mg/kg as commonly suggested and well tolerated [31, 32], a regimen that achieves peak plasma levels of approximately 1.5 µg/mL and a terminal half-life of approximately 44 h, mirroring efficacious exposures in patients while maintaining good tolerability [33]. The anti-mouse PD-L1 antibody (aPD-L1) was given 200 μg per mouse (which approximately 10 mg/kg) via intraperitoneal route three times a week as recommended [34].

Drug administration was followed by irradiation for 1 h [35]. Serum samples were collected one day before treatment, during the second week of treatment, and in the third week (post-treatment) to measure IFN-γ levels, as previously described. After euthanasia, the tumors were excised for further analysis, including flow cytometry for quantifying TILs and immunohistochemistry (IHC).

### Immunohistochemistry staining

Tumor specimens from mice were initially rinsed with phosphate-buffered saline (PBS) and subsequently fixed in 4% formaldehyde (Mallinckrodt Chemical Co., St. Louis, MO, USA) diluted in PBS. After fixation, the specimens were embedded in paraffin. Sections of 6 µm thickness were then prepared from the paraffin-embedded tissues. The sections were deparaffinized before application of primary antibodies for IHC staining [36]. The primary antibodies used were against CD4 (HS-360 117, Sysy), CD8 (HS-361 003, Sysy), monocyte chemotactic protein-1 (MCP-1, a marker of M1 macrophages; orb323291, Biorbyt) [37], CD80 (a marker of M1 macrophages; HPA050092, Sigma-Aldrich) [38], inducible nitric oxide synthase (iNOS, a marker of M1 macrophages; PA3-030A, ThermoFisher) [39], and CD206 (a marker of M2 macrophages; ab64693, Abcam) [40]. Antigens were visualized using an indirect immunoperoxidase technique following the manufacturer’s protocol.

### Statistical analysis

All experiments were performed independently at least three times to ensure reproducibility. Results are expressed as the mean ± standard deviation (SD). Details of the statistical tests used for each dataset are provided in the corresponding figure legends. Statistical significance was set at *P* < 0.05, with significance levels indicated as follows: * *P* < 0.05, ** *P* < 0.01, *** *P* < 0.001, and **** *P* < 0.0001. Error bars in the figures denote SD. Statistical analyses and graph generation were conducted using GraphPad Prism (version 9.5.1, GraphPad Software, Inc., Windows).

## Results

### CDK4/6 inhibitors have anti-tumor effects in TNBC cell lines

To evaluate the effectiveness of the CDK4/6 inhibitor, abemaciclib, in TNBC cell lines, we assessed the viability of MDA-MB-231, MDA-MB-453, and MDA-MB-468 cells treated with different doses of abemaciclib. The IC_50_ values for abemaciclib were found to be 12.15, 2.86, and 8.01 µM for MDA-MB-231, MDA-MB-453, and MDA-MB-468 cells, respectively; the corresponding cell survival curves are presented in Supplementary Fig. 1.

CDK4/6 plays a critical role in cell cycle progression from the G1 to the S phase by phosphorylating RB, subsequently releasing the E2F transcription factor [41]. We investigated RB expression in five cell lines using western blot analysis (Supplementary Fig. 2). Cells were treated with either a control, 4 Gy of RT, 50 nM abemaciclib, or a combination of RT and abemaciclib. The levels of RB and phosphorylated RB (p-RB) were assessed after 24 h of treatment. All cell lines demonstrated RB expression, our findings revealed that abemaciclib effectively inhibited phosphorylation of RB, as evidenced by reduced levels of p-RB, whereas RT did not significantly alter p-RB levels (Supplementary Fig. 2).

### CDK4/6 inhibitor sensitizes TNBC cells to ionizing irradiation

**Fig. 1:**
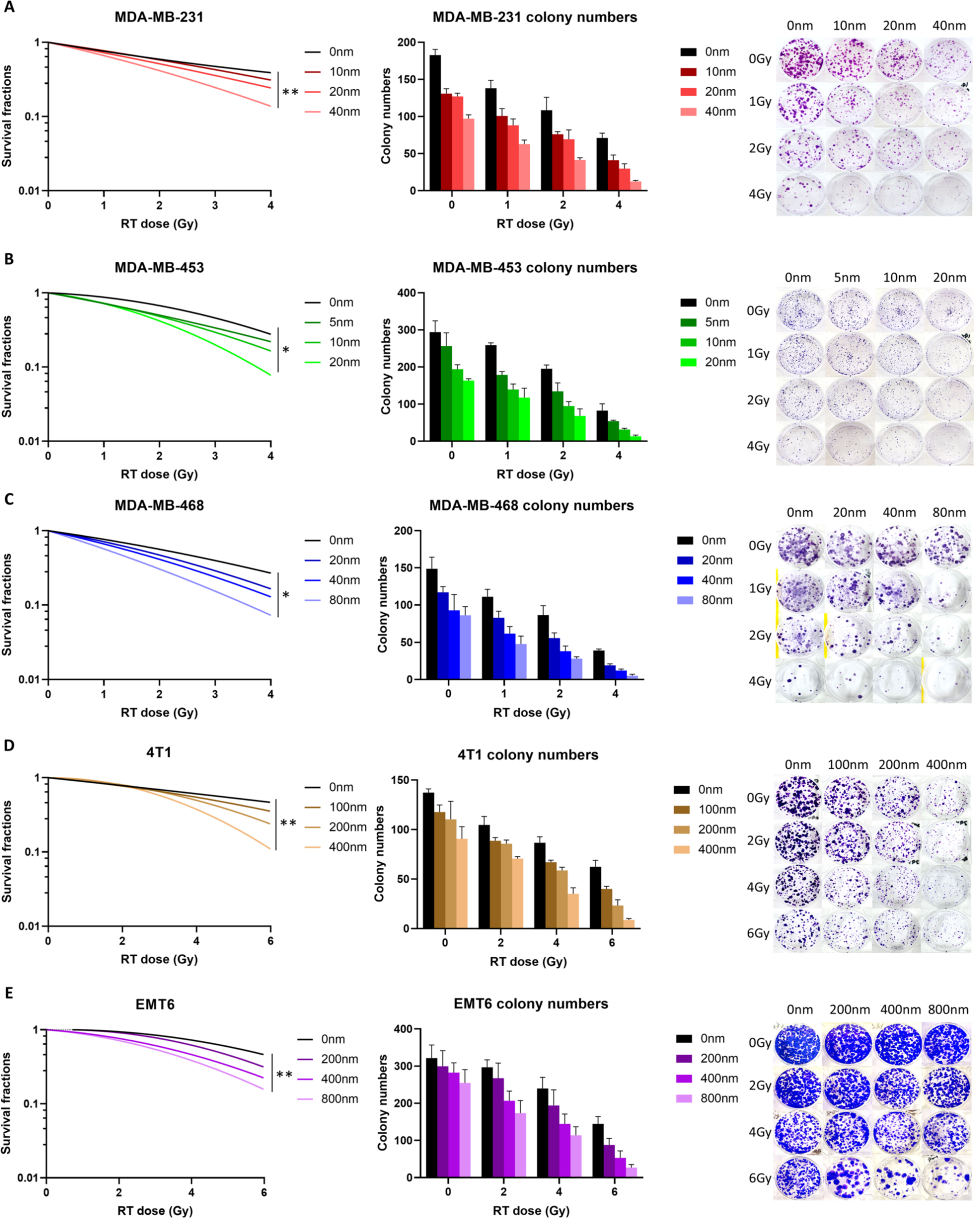
Abemaciclib synergizes with radiation therapy (RT) to significantly reduce the survival of triple-negative breast cancer cell lines, as demonstrated through colony-formation assays. Clonogenic assay results for (**A**) MDA-MB-231, (**B**) MDA-MB-453, (**C**) MDA-MB-468, (**D**) 4T1, and (**E**) EMT6 cells treated with varying doses of abemaciclib (0–40 nM for MDA-MB-231, 0–20 nM for MDA-MB-453, 0–80 nM for MDA-MB-468, 0–400 nM for 4T1, and 0–800 nM for EMT-6) and RT (0–6 Gy). Abemaciclib was administered 1 h before RT. Colonies were fixed, stained, and counted after 7–14 days. The left columns display the surviving fractions of all five cell lines fitted to a linear quadratic model. The middle columns represent the average number of colonies and their standard deviations. The right columns show photographs of the colonies, illustrating the effects of combining various RT doses with different concentrations of abemaciclib on the five TNBC cell lines. All experiments were conducted at least three times. Statistical analysis was conducted using two-way analysis of variance (ANOVA) with Tukey’s post-hoc test. Significance levels: *P < 0.05; **P < 0.01

Radiosensitization effect have been investigated in luminal type breast cancer and other cancer types as well [41, 42]. To confirm the combined benefits of RT and CDK4/6 inhibitors in TNBC treatment, the radiosensitizing effects of abemaciclib on TNBC cell lines were tested. A clonogenic assay was performed to assess whether abemaciclib can enhance the sensitivity of TNBC cell lines to RT. MDA-MB-231, MDA-MB-453, MDA-MB-468, 4T1 and EMT6 cells were pretreated with various doses of abemaciclib (0–80 nM for MDA-MB-231, MDA-MB-453, and MDA-MB-468; 0–400 nM for 4T1, and 0–800 nM EMT6) for 1 h before exposure to different doses of RT (0–4 Gy for MDA-MB-231, MDA-MB-453, and MDA-MB-468; 0–6 Gy for 4T1 and EMT6). A significant synergistic effect between abemaciclib and RT was observed in all five cell lines, demonstrating a dose-dependent radiosensitization effect, as illustrated in Fig. 1. Colony survival data were plotted using a linear quadratic model via nonlinear regression, as detailed in Fig. 1A–E. Higher dose of abemaciclib demonstrated significantly better radiosensitization effect across five cell lines (*P* < 0.05).

### Radiotherapy increases cell surface PD-L1 expression in TNBC cells

**Fig. 2:**
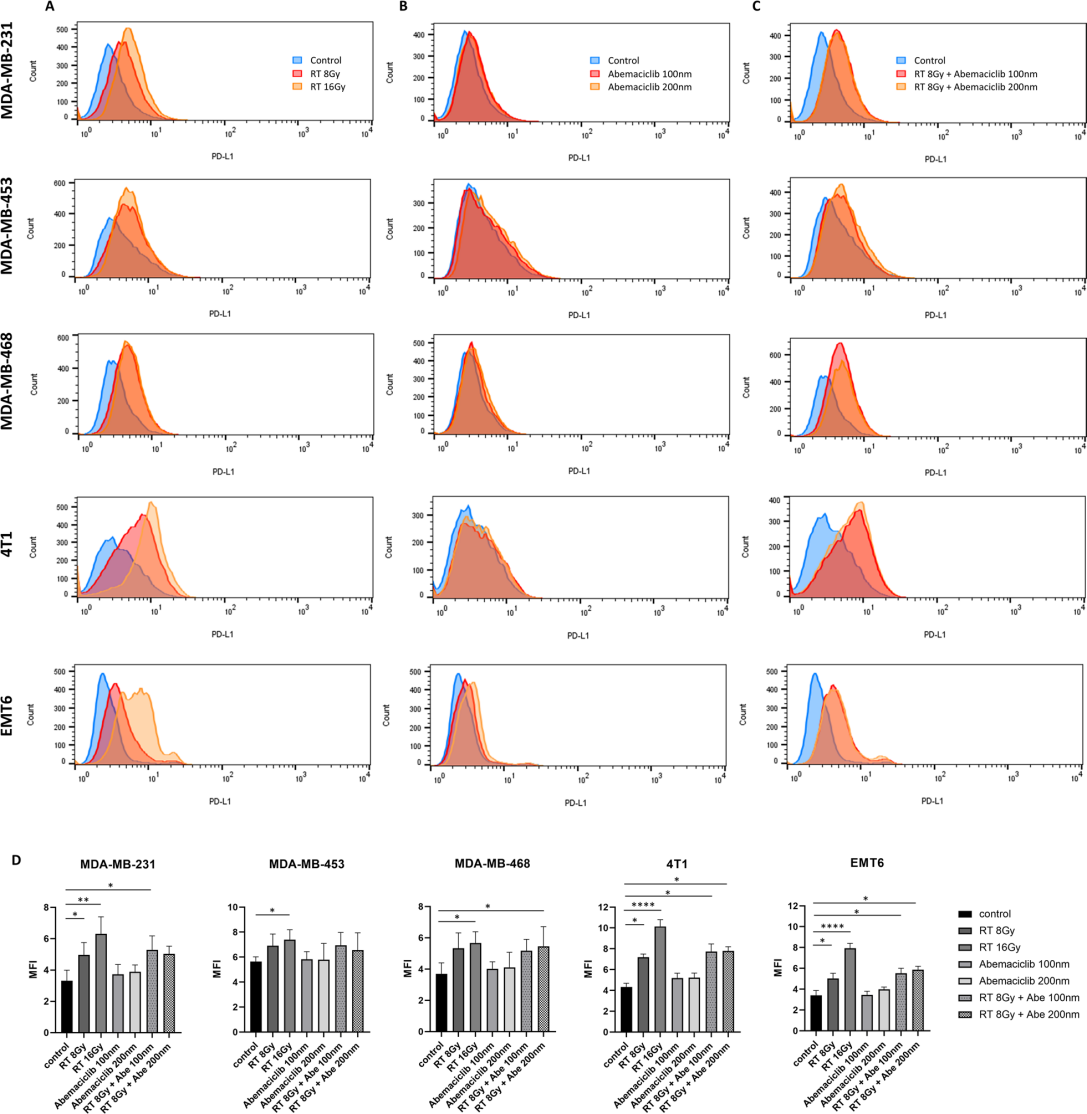
Surface PD-L1 expression in triple negative breast cancer cell lines under different doses of RT and abemaciclib. MDA-MB-231, MDA-MB-453, MDA-MB-468, 4T1, and EMT6 cells were treated with (**A**) different doses of RT (8 Gy, 16 Gy, or no RT), (**B**) different doses of abemaciclib (100 nM, 200 nM, or without abemaciclib), or (**C**) RT (8 Gy) in combination with abemaciclib at 100 nM or 200 nM, or without any treatment. The cells were collected 24 h after treatment. Subsequently, all cells were stained with an anti-PD-L1 antibody and analyzed using a LSR II flow cytometer (BD Biosciences, San Diego, CA). **D** Mean fluorescence intensity (MFI) bar charts quantify PD-L1 expression levels under each condition and presented as mean ± standard deviation. The difference in MFI values was assessed by an unpaired two-tailed t-test. All experiments were conducted three times. Significance levels: *P < 0.05; **P < 0.01; ***P < 0.001, ****P < 0.0001

Both RT and CDK4/6 inhibitors have been recognized for their immunomodulatory effects [43–45], prompting investigations to determine whether their combination can enhance the efficacy of anti-PD-L1 therapy. The present study evaluated the influence of RT and abemaciclib, both alone and in combination, on the expression of PD-L1 on the cell surface. MDA-MB-231, MDA-MB-453, MDA-MB-468, 4T1, and EMT6 cells were treated with varying doses of RT (0, 8, or 16 Gy), abemaciclib (0, 100, or 200 nM) alone, or a combination of 8 Gy RT and abemaciclib pretreatment (100 or 200 nM) 1 h before RT. Our findings revealed that RT at 8 and 16 Gy notably increased PD-L1 expression in all five cell lines (Fig. 2A and D). In contrast, abemaciclib alone did not affect the surface expression of PD-L1 (Fig. 2B and D). Combining RT with abemaciclib mirrored the effects of RT alone on PD-L1 expression, suggesting that abemaciclib did not alter the RT-induced modulation of PD-L1 expression (Fig. 2C and D). These results indicate that the combination of RT and abemaciclib did not enhance or diminish the individual effects of RT on PD-L1 expression levels.

### CDK4/6 inhibitor combined with RT enhances anti-PD-L1 efficacy in vivo of TNBC

**Fig. 3:**
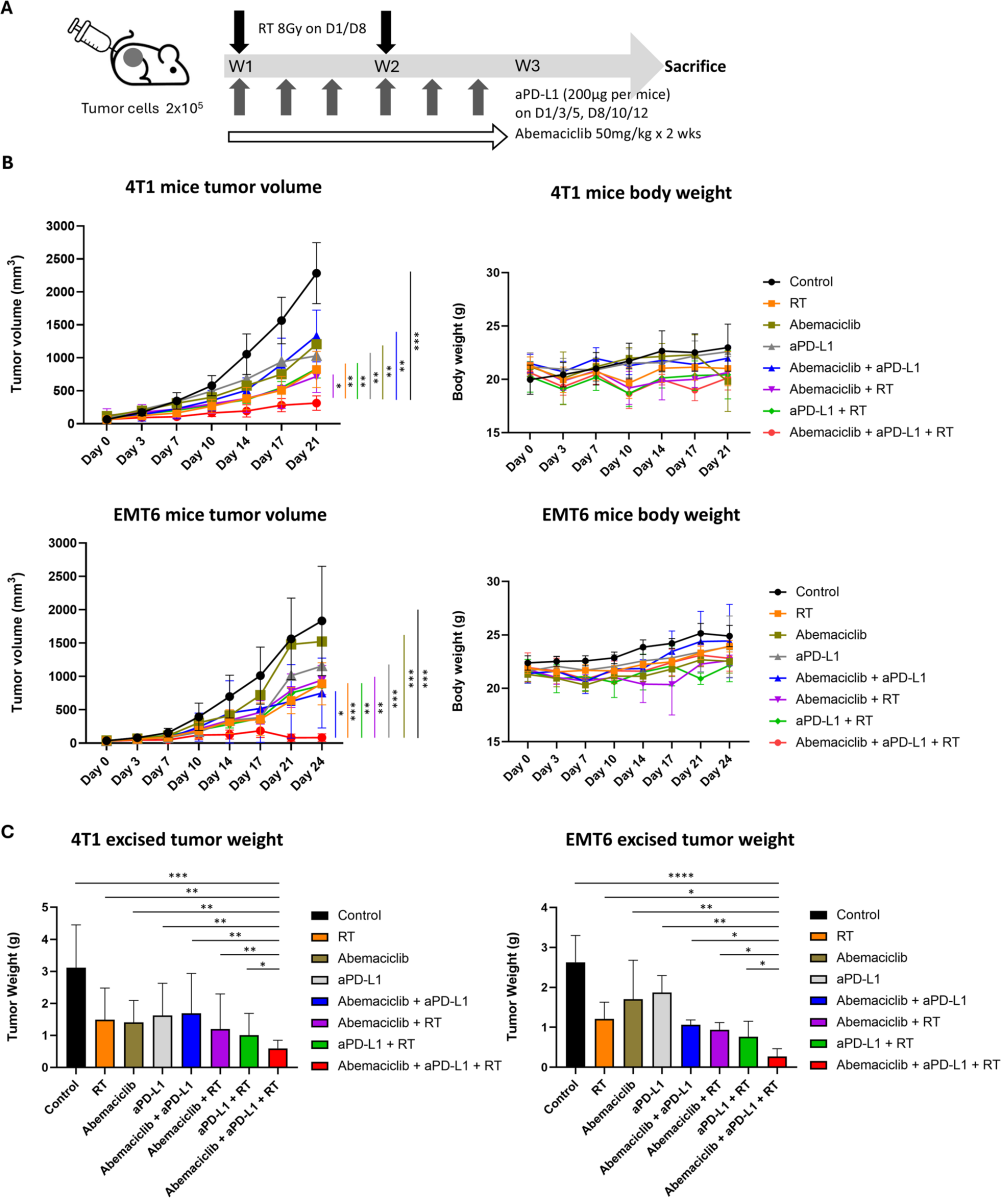
Antitumor effect of different combinations of RT, abemaciclib, and anti-PD-L1 antibody (aPD-L1) in vivo. **A** 4T1 and EMT6 mouse models were used with female BALB/c mice for the in vivo study. The schematic diagram of the therapeutic protocol is shown. Eight mice were included in each group. **B** The tumor volume and body weight of the two mouse models treated with RT, abemaciclib, aPD-L1, or their dual and triple combinations. The tumor volume and body weight were measured twice a week during the treatment period. The tumor volume difference was assessed by two-way analysis of variance (ANOVA) with mixed-effect analysis. **C** After euthanasia, the tumors were excised and weighed. The excised tumor weight difference was assessed by an unpaired two-tailed t-test. Significance levels: *P < 0.05; **P < 0.01; ***P < 0.001, ****P < 0.0001

Although CDK4/6 inhibitors do not significantly elevate cell surface PD-L1 expression like RT, they possess immunomodulatory effects that can potentiate immunotherapy [31, 43, 44]. Additionally, high-dose RT is widely acknowledged for boosting immune responses and enhancing the efficacy of immunotherapy [19, 45]. In the present study, we aimed to evaluate the synergistic effects of combining RT with CDK4/6 inhibitors and anti-PD-L1 antibodies on tumor suppression in vivo using 4T1 and EMT6 immunocompetent mouse models. Mice were divided into eight treatment groups: control, RT alone, abemaciclib alone, aPD-L1 alone, abemaciclib combined with aPD-L1, abemaciclib combined with RT, aPD-L1 combined with RT, and abemaciclib combined with aPD-L1 and RT. The various treatment strategies are detailed in Fig. 3A. Over a two-week treatment period, tumor sizes were monitored twice a week. There is no significant difference between mice weights during and after treatment in different treatment groups (Fig. 3B). The mice were euthanized after one to two weeks of follow-up till tumor volume exceed 1500–2000 mm^3^. The tumors were excised and their volumes were analyzed. The data are presented in Fig. 3B and C. We found that the group receiving the combination treatment of abemaciclib, aPD-L1, and RT exhibited a significant reduction in tumor growth compared to all other groups (all, *P* < 0.05). Additionally, tumors excised from the triple combination group showed significantly lower weight than those from the control group in both 4T1 (*P* < 0.001) and EMT6 murine models (*P* < 0.0001), as depicted in Fig. 3C. When compared with other groups, the tumor weights of the triple combination group were still lower (all, *P* < 0.05). We found that no significant difference was observed in tumor growth between single-modality treatments (RT alone, abemaciclib alone, or aPD-L1 alone) and dual treatments such as abemaciclib combined with aPD-L1, abemaciclib combined with RT, or aPD-L1 combined with RT. This highlights the enhanced efficacy of the triple combination in comparison with single or dual treatments. These findings suggest that the combination of abemaciclib and RT significantly enhanced the antitumor efficacy of aPD-L1 in comparison with the other treatment arms. We examined lung metastases and found no significant differences among the treatment groups. This observation suggests that while the treatments effectively control primary tumor growth, their impact on metastatic progression requires further investigation.

### CDK4/6 inhibitor, RT, and anti-PD-L1 increase circulating IFN-γ levels

We also monitored the changes in immune-related cytokines throughout the treatment period in all the groups. IFN-γ, a pivotal cytokine in the immune response, serves as a biomarker that reflects the efficacy of immunotherapy. IFN-γ is known to be upregulated by checkpoint inhibitors, highlighting their role in immune surveillance and therapeutic responses [46]. Therefore, we evaluated the circulating IFN-γ levels in our 4T1 mouse model.

**Fig. 4:**
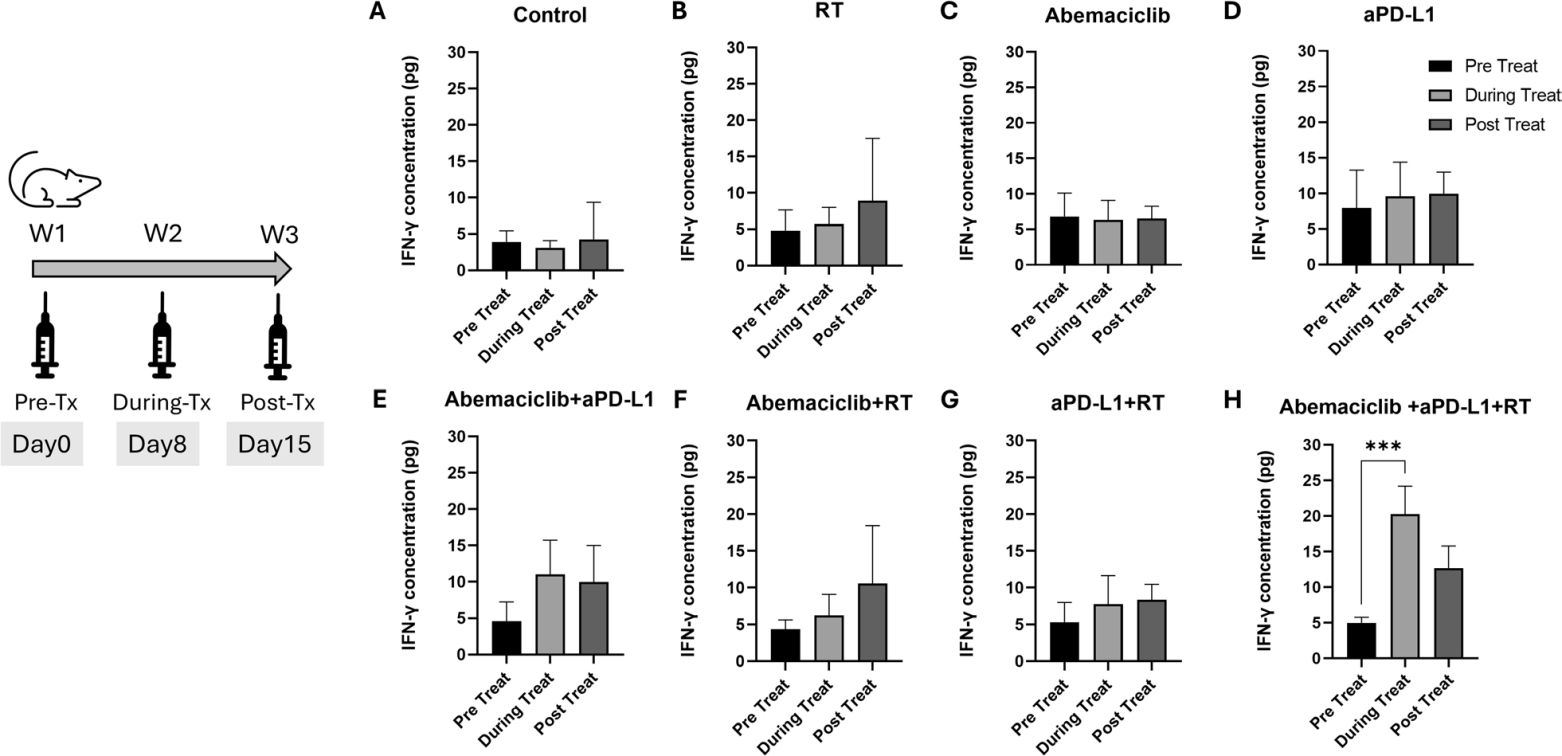
Circulating IFN-γ levels before, during, and after treatment with different combinations of RT, abemaciclib, and anti-PD-L1 antibody (aPD-L1). Blood samples from the 4T1 mouse model were obtained one day before treatment, during treatment (day 8), and after treatment (day 15). IFN-γ levels were measured using enzyme-linked immunosorbent assays. The IFN-γ levels of the (**A**) control, (**B**) RT alone, (**C**) abemaciclib alone, (**D**) aPD-L1 alone, (**E**) abemaciclib combined with aPD-L1, (**F**) abemaciclib combined with RT, (**G**) aPD-L1 combined with RT, and (**H**) abemaciclib combined with aPD-L1 and RT groups are expressed as mean ± standard deviation. The experiments were repeated three times independently. The differences were assessed by an unpaired two-tailed t-test. Significance levels: *P < 0.05; **P < 0.01; ***P < 0.001

Blood samples were collected from mice before, during, and after treatment, and IFN-γ levels were measured using ELISA. Figure 4 displays the changes in circulating IFN-γ levels before, during, and after treatment for each treatment group. In the control (Fig. 4A), abemaciclib (Fig. 4C), and aPD-L1 (Fig. 4D) groups, no differences were observed in IFN-γ levels before, during, or after treatment. In the RT alone (Fig. 4B), abemaciclib combined with aPD-L1 (Fig. 4E), abemaciclib combined with RT (Fig. 4F), and aPD-L1 combined with RT groups (Fig. 4G), the IFN-γ levels exhibited slight increase during and post-treatment, although these changes did not reach statistical significance. In the triple combination group (Fig. 4H), circulating IFN-γ levels significantly increased during treatment (*P* < 0.001) and then decreased post-treatment.

### CDK4/6 inhibitor, RT, and anti-PD-L1 modulate tumor microenvironment by recruiting CD4 + T cells, CD8 + T cells, and M1 type macrophages

**Fig. 5:**
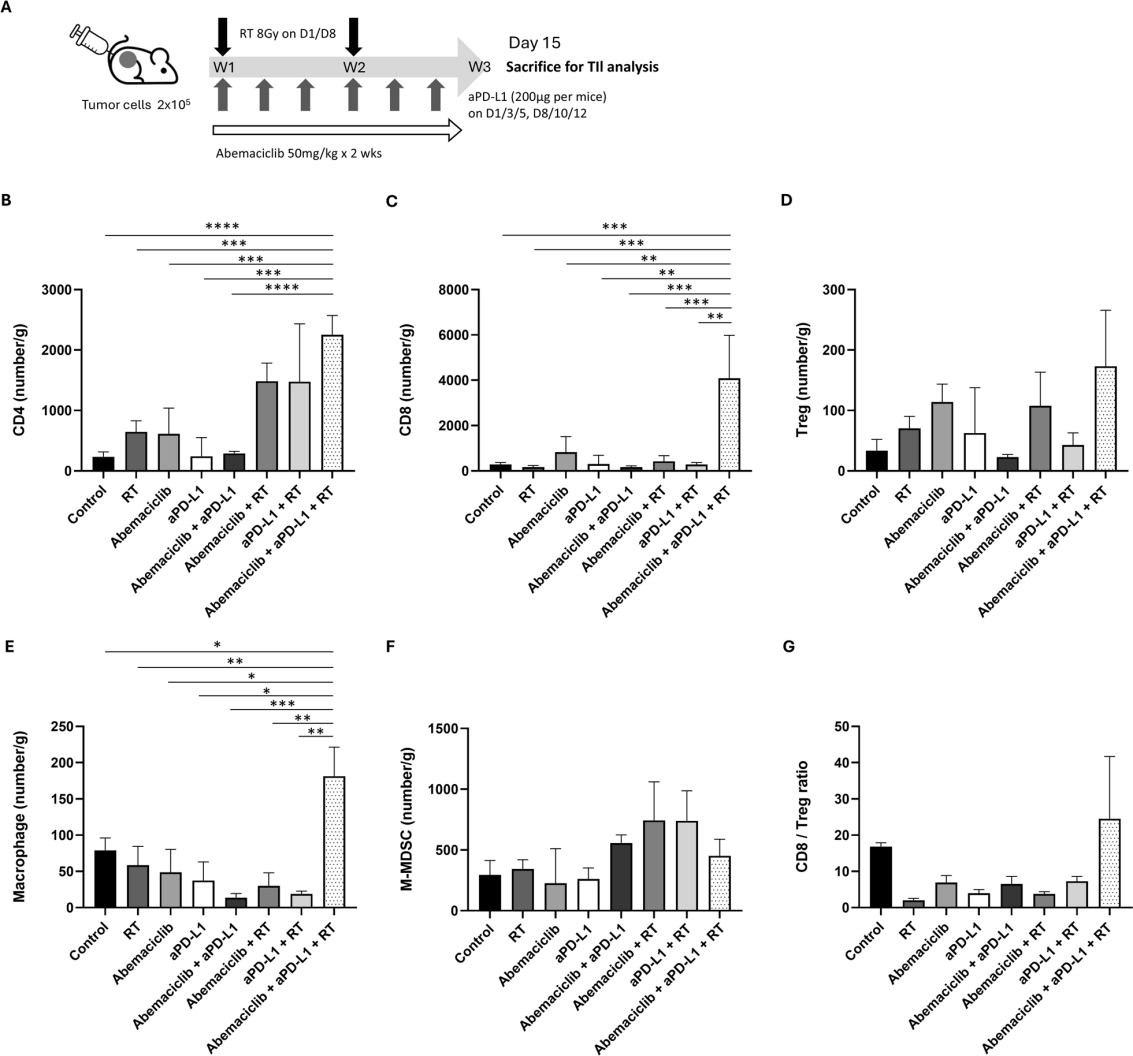
Tumor-infiltrating lymphocyte (TIL) analysis in tumors excised from an in vivo mouse model receiving treatment with different combinations of abemaciclib, RT, and anti-PD-L1 antibody (aPD-L1). **A** TILs were analyzed from the excised tumors from a 4T1 mouse model subjected to different treatment conditions, including control, RT alone, abemaciclib alone, aPD-L1 alone, abemaciclib combined with aPD-L1, abemaciclib combined with RT, aPD-L1 combined with RT, and abemaciclib combined with aPD-L1 and RT. The tumors were collected after the two-week treatment (on day 15). **B** The CD4 + T cells, **C** CD8 + T cells, **D** regulatory T cells (Tregs), **E** macrophages, and **F** the monocytic-myeloid-derived suppressor cells (M-MDSC) were quantified and displayed as number of cells per gram. The CD8 to Treg ratio is demonstrated (**G**). All data are presented as mean ± standard deviation. The experiments were repeated three times. The differences in TILs in different treatment conditions were assessed by an unpaired two-tailed t-test. Significance levels: *P < 0.05; **P < 0.01; ***P < 0.001; ****P < 0.0001

Our findings demonstrated that the triple combination of abemaciclib, aPD-L1, and RT enhanced both antitumor effect and circulating IFN-γ levels. To further understand how this treatment combination affects the tumor microenvironment, we evaluated the changes in immune cell populations across different treatment groups. Using the same 4T1 tumor mouse model with the same treatment schedule, we excised tumors right after treatment (Fig. 5A) to assess TILs, including CD4 + T cells, CD8 + T cells, macrophages, monocytic*-*myeloid-derived suppressor cells (M-MDSCs)*,* and regulatory T cells (Tregs) under various treatment conditions. The gating strategies are illustrated in Supplementary Fig. 3.

We observed that the triple combination group (abemaciclib combined with aPD-L1 and RT) exhibited significantly increased infiltration of CD4 + T cells compared to the control (*P* < 0.0001), RT alone (*P* < 0.001), abemaciclib alone (*P* < 0.001), aPD-L1 alone (*P* < 0.001), and abemaciclib combined with aPD-L1 groups (*P* < 0.0001), as shown in Fig. 5B. Additionally, CD8 + T cells were significantly increased in the triple combination group compared to all other groups (All *P* < 0.01), as shown in Fig. 5C.

Macrophage numbers also increased after the triple combination treatment, as depicted in Fig. 5E. This increase was significant compared to that in the control (*P* < 0.05), RT alone (*P* < 0.01), abemaciclib alone (*P* < 0.05), aPD-L1 alone (*P* < 0.05), abemaciclib combined with aPD-L1 (*P* < 0.001), abemaciclib combined with RT (*P* < 0.01), and aPD-L1 combined with RT (*P* < 0.01) groups. Conversely, no significant differences were observed in Tregs, M-MDSCs, or the CD8 to Treg ratio among the treatment groups (Fig. 5D, F, and G).

To confirm the changes in the tumor microenvironment under different treatment conditions, we performed IHC staining on the resected tumors from 4T1 and EMT6 mouse models. We assessed the expression of PD-L1 in tumor cells of murine EMT6 cells (in vivo model) treated with different modalities via IHC analysis. Our findings revealed that PD-L1 expression was significantly upregulated in the tumor cells of the RT alone, abemaciclib with RT, aPD-L1 with RT, and triple combination groups in comparison with the control group (*P* < 0.05, *P* < 0.001, *P* < 0.0001, and *P* < 0.001, respectively). In contrast, no changes in PD-L1 expression were observed in the abemaciclib, aPD-L1, and abemaciclib plus aPD-L1 groups (Supplementary Fig. 4).

**Fig. 6:**
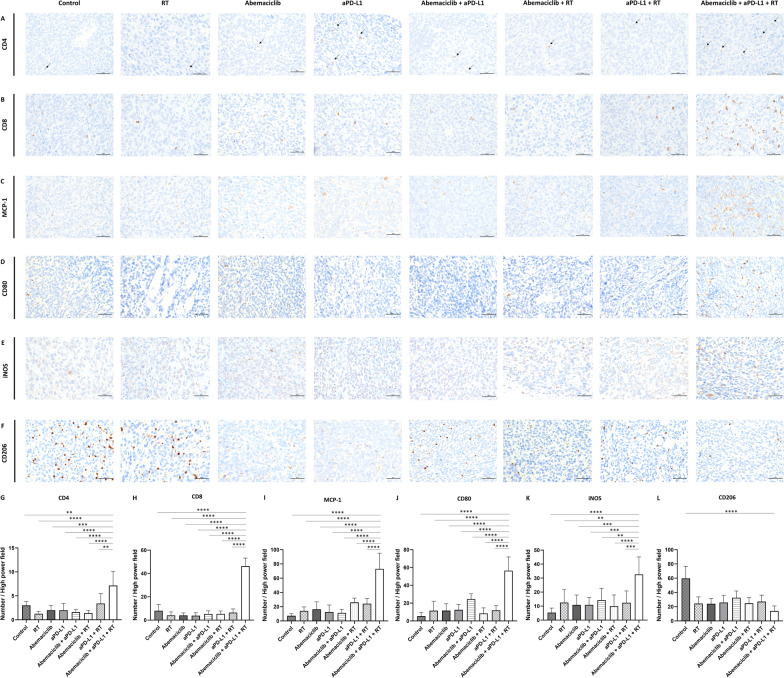
Immunohistochemical staining of CD4 + T cells, CD8 + T cells, and MCP-1-, CD80-, iNOS- and CD206-positive macrophages in tumors excised from an in vivo model after treatment with different combinations of abemaciclib, RT, and anti-PD-L1 antibody (aPD-L1). Immunohistochemical staining of the resected mouse tumors from 4T1 was performed for CD4, CD8, MCP-1, CD80, and CD206. The upper panel shows (**A**) CD4 + , (**B**) CD8 + , (**C**) MCP-1 + , (**D**) CD80 + , (**E**) iNOS + , and (**F**) CD206 + cells in the different treatment groups, including the control, RT alone, abemaciclib alone, aPD-L1 alone, abemaciclib combined with aPD-L1, abemaciclib combined with RT, aPD-L1 combined with RT, and abemaciclib combined with aPD-L1 and RT groups. The scale bar represents 50 μm. The lower panel shows the quantification of the average number of CD4 + T cells (**G**), CD8 + T cells (**H**), MCP-1 + (**I**), CD80 + (**J**), iNOS + (**K**), and CD206 + (**L**) cells within the resected tumor in the high-power field of the image. The differences were assessed by an unpaired two-tailed t-test. Significance levels: **P < 0.01; ***P < 0.001; ****P < 0.0001

In 4T1 mouse model, we found that CD4 + T cell numbers were higher in the triple combination group than in the other five groups, including the control (*P* < 0.01), RT alone (*P* < 0.0001), abemaciclib alone (*P* < 0.001), aPD-L1 alone (*P* < 0.0001), abemaciclib combined with aPD-L1 (*P* < 0.0001), abemaciclib combined with RT (*P* < 0.0001), and aPD-L1 combined with RT (*P* < 0.01) groups (Fig. 6A and G). In addition, the triple combination group had a significantly higher number of CD8 + T cells than the other five groups (Fig. 6B and H, *P* < 0.0001 compared to all other groups). Tumor-associated macrophages can exhibit a pro-inflammatory M1 or immunosuppressive M2 phenotype. To distinguish the subtypes of increased macrophages observed in the tumor-infiltrating lymphocytes analysis, we used MCP-1, CD80, and iNOS as M1 markers [37–39], while CD206 as an M2 marker [40]. We found that the number of MCP-1 positive cells was higher in the triple combination group than in the other five groups (*P* < 0.0001) as in Fig. 6C and I. Similarly, the number of CD80-positive and iNOS-positive cells was also elevated in the triple combination group compared to the other treatment groups (Fig. 6D, E, J, and K) (all *P* < 0.01), further supporting an increase in M1-type macrophages with this treatment. The number of CD206-positive cells, indicative of M2 macrophages, decreased in the triple combination group in comparison with that in the control group (*P* < 0.0001) (Fig. 6E and J). This shift in macrophage populations, characterized by an increase in pro-inflammatory M1 macrophages (MCP-1 +, CD80 +, and iNOS +) and a decrease in immunosuppressive M2 macrophages (CD206 +), suggests that the triple combination treatment effectively reprogrammed the tumor microenvironment toward an immunostimulatory state. We performed IHC staining and analysis of the EMT6 mouse model and observed consistent results (Supplementary Fig. 5). This further supports the robust effect of triple combination treatment in modulating the tumor microenvironment in different TNBC mouse models.

## Discussion

Poor prognosis and lack of specific treatment targets make TNBC treatment difficult. While advancements in molecular diagnostics have led to targeted therapies, such as poly-(ADP-ribose) polymerase (PARP) inhibitors and antibody–drug conjugates [47], these treatments have not significantly impacted clinical practice due to the heterogeneity of TNBC.

CDK4/6 inhibitors have demonstrated synergistic effects with anti-estrogen therapies. For example, palbociclib combined with letrozole prolonged survival in ER-positive, human epidermal growth factor receptor 2 (HER2)-negative breast cancer, establishing this combination as a first-line therapy [48]. The efficacy of CDK4/6 inhibition depends on the functional RB phosphorylation [49]. As RB is frequently lost in TNBC tumor samples [50], CDK4/6 inhibitors are generally thought to be ineffective against these cancers. However, preclinical studies paint a different picture. Asghar et al. conducted a detailed analysis using a panel of 12 TNBC cell lines, demonstrating that luminal androgen receptor-positive TNBC cells were more responsive to CDK4/6 inhibitors (palbociclib and ribociclib) than basal-like cell lines such as MDA-MB-468 [13]. In our study, all TNBC cell lines (MDA-MB-231, MDA-MB-453, MDA-MB-468, 4T1, and EMT6) were sensitive to abemaciclib, which is 5.5 times more potent than palbociclib in terms of cell killing efficiency [51].

The radiosensitizing effect of CDK4/6 inhibitors has been well-documented [52, 53], primarily by impairing DNA repair mechanisms. DNA damage, particularly double-strand breaks, triggers a complex post-translational network known as the DNA damage response. Previous studies have shown that combining CDK4/6 inhibitors with ionizing radiation delays DNA repair and increases γH2AX levels 24 h post-radiation [21]. Pesch et al. showed that RB expression in tumor cells dictates the response to the CDK4/6 inhibitor palbociclib combined with RT in TNBC [54]. In this study, the radiosensitizing effect of abemaciclib was demonstrated in three human and two murine TNBC cell lines. All five cell lines expressed RB, and treatment with abemaciclib significantly reduced p-RB levels. These findings are consistent with those reported by Pesch et al., highlighting the critical role of RB in mediating the response to CDK4/6 inhibitors in combination with RT in TNBC [54].

CDK4/6 inhibitors have been reported to enhance the immune response in several ways, including promoting cytotoxic T-cell activity, enhancing tumor antigen presentation, and recruiting innate immune cells [16]. Additionally, CDK4/6 inhibitors activate the expression of endogenous retroviral elements in tumor cells, stimulate the production of type III interferons, and further boost antigen presentation in tumors [55]. CDK4/6 inhibitors also significantly limit the abundance of Tregs [44], collectively enhancing the immune response and antitumor effects. Building on these mechanistic data, we selected an oral abemaciclib dose of 50 mg/kg once daily for 14 days, combined with two weekly fractions of 8 Gy RT based on robust pre-clinical evidence. Torres-Guzmán et al. showed that 50 mg/kg abemaciclib yields sustained tumor regression [32], Flint et al. confirmed favorable tissue penetration at similar abemaciclib exposures [33], while Buchwald et al*.* and Dewan et al*.* [29, 30] demonstrated that hypofractionated RT doses of 8–10 Gy, administered two to three times, optimally prime dendritic cells and synergize with immune checkpoint blockade. Collectively, these findings support the use of a pharmacologically active yet well-tolerated regimen.

In a recent clinical trial published by Rugo et al. [56], combining abemaciclib with pembrolizumab showed promising activity but marked grade ≥ 3 toxicities in patients with hormone receptor-positive and HER2-negative metastatic breast cancer. In view of this finding, we purposely used sub-IC₅₀ concentrations (100–200 nM) and the lowest effective murine dose (50 mg/kg QD) of abemaciclib [22, 32, 33]. These attenuated exposures of abemaciclib still sensitized TNBC cells to RT and—when combined with two fractions of 8 Gy RT and aPD-L1—increased tumor control without detectable toxicity. These results suggest that any future clinical trial combining CDK4/6 inhibition with RT and PD-L1 blockade should initiate dose exploration at the lowest feasible abemaciclib level and escalate only as safety permits.

Some studies have shown that CDK4/6 inhibitors upregulate PD-L1 expression [57], suggesting that combining CDK4/6 inhibitors with ICIs could be beneficial. Deng et al. showed that combining ICIs with CDK4/6 inhibitors effectively reduced tumor proliferation in an in vivo model, highlighting the potential of this therapeutic strategy [43]. However, the regulation of PD-L1 by CDK4/6 blockade is highly context-dependent. In this study, abemaciclib did not upregulate PD-L1 expression in any TNBC cell line. The impact of CDK4/6 inhibitors on PD-L1 expression has been inconsistent across studies. Murine 4T1 and EMT6 cells are RB-proficient and exhibit relatively high basal PD-L1, a setting in which recent mechanistic work by Shrestha et al. showed that CDK4/6 inhibition can suppress rather than induce PD-L1 through a phosphorylated RB–E2F1–SPOP axis [58]. In contrast, PD-L1 upregulation is more frequently observed in ER-positive or RB-deficient models, where transcriptional activation outweighs SPOP-mediated degradation [57]. Additional variables—such as solvent pH, treatment duration, and glycolytic acidification—can further shift the balance between PD-L1 synthesis and turnover [58]. The interaction between CDK4/6 inhibitors and speckle-type POZ proteins may also contribute to this discrepancy [57].

The success of immunotherapy has improved the survival rate of patients with TNBC. However, ICIs alone appear to be less effective than combination therapy. The Keynote 086 trial showed that pembrolizumab benefits only a small proportion of patients with advanced-stage PD-L1-positive TNBC [59]. Similarly, Emens et al*.* reported a limited effect of the anti-PD-L1 agent, atezolizumab, in patients with metastatic TNBC [5]. Studies have indicated that combining immunotherapy with other antitumor agents is more effective than immunotherapy alone [60]. The rationale behind this combination is the potentiation of the immune system by other treatments such as chemotherapy, RT, and targeted therapies. Combining chemotherapy and immunotherapy successfully prolonged overall survival in patients with metastatic TNBC and a PD-L1 combined positive score of 10 or more [61]. The combination of pembrolizumab and chemotherapy also benefits patients with early-stage TNBC [7]. However, PD-L1 expression is not a reliable predictor of checkpoint inhibitor response, as seen in KEYNOTE-522 [7], where PD-L1 status did not predict the pathological complete response rate. Although combination therapy improved survival in the KEYNOTE-355 trial [61], approximately half of the patients with TNBC did not respond to immunotherapy combined with chemotherapy. This suboptimal response rate prompted us to develop new combinations of immunotherapies for treating TNBC. Therefore, the main challenge is to explore new treatment strategies for enhancing the tumor response of TNBC to immunotherapy and reversing the resistance of responders to ICIs.

As previously mentioned, CDK4/6 inhibitors and RT have been reported to independently enhance immune responses. Multiple pre-clinical studies have indicated that these three treatments (RT, abemaciclib, and aPD-L1) relieve distinct, non-overlapping bottlenecks in the cancer-immunity cycle. RT supplies tumor antigens and type I IFN/STING signaling but simultaneously induces PD-L1 expression and recruits suppressive myeloid cells, limiting its stand-alone efficacy [62]. CDK4/6 inhibition with abemaciclib augments antigen presentation, upregulates IFN-stimulated genes, and promotes inflammation within the tumor microenvironment, thereby priming CD8⁺ T-cell infiltration; however, this inflammatory response may still be checked by PD-L1 upregulation. Checkpoint blockade removes that brake yet remains ineffective in “cold” tumors unless antigen release and innate priming have already occurred [52]. When all three modalities are combined, the antigen supply (RT), inflammatory conditioning (abemaciclib), and checkpoint relief (aPD-L1) act together, resulting in non-linear tumor control. Our data align with this model: RT + aPD-L1 doublet did not exceed RT alone, showing comparable circulating IFN-γ, CD4⁺/CD8⁺ T-cell density and M1 macrophage levels (Figs. 4, 5, 6). The RT + abemaciclib doublet produced only a modest, non-significant rise in IFN-γ and CD8⁺ T-cell infiltration (Figs. 4, 5, 6), suggesting that residual PD-L1 signaling may still have dampened effector activity. The abemaciclib + aPD-L1 doublet lacked the immunogenic boost provided by RT; therefore, it may not have generated sufficient tumor antigen release to prime a stronger adaptive response, which could explain its limited benefit over monotherapy. Only the triple combination raised IFN-γ and immune-cell infiltration and significantly delayed tumor growth (Figs. 4, 5, 6). A recent independent study reached a similar conclusion: adding abemaciclib converted a partially active RT + aPD-L1 doublet into a markedly effective triplet by further inflaming the tumor microenvironment and enhancing CD8⁺ infiltration [63]. Taken together, these findings explain the apparent non-linearity and underscore the necessity of simultaneously addressing all three immune evasion axes.

Our study is the first to combine these two immune-responsive potentiators to improve immunotherapeutic outcomes in TNBC. We used two immunocompetent mouse models in this study: the 4T1 model, which was selected for its poor immunogenicity and high metastatic potential, reflecting the characteristics of advanced human TNBC [24], and the EMT6 model, which was chosen for its relatively high immunogenicity [64]. Our findings showed that the triple combination of abemaciclib, RT, and aPD-L1 significantly increases circulating IFN-γ levels more than other treatment strategies. IFN-γ is known to stimulate antigen-presenting cells, inhibit the function of MDSCs, and induce a phenotypic switch in tumor-associated macrophages from the M2 to M1 subtype [46]. Although previous studies have indicated that CDK4/6 inhibitors suppress Tregs, this effect was not clearly observed in our study, likely due to the opposing influence of RT [65], which has been reported to increase Treg numbers and recruit them to the tumor site. As shown in Fig. 5D, RT alone, RT combined with abemaciclib, and RT combined with abemaciclib and aPD-L1 increased Treg numbers, although the differences were not significant. Neither abemaciclib nor RT combined with aPD-L1 increased CD4 + T cells, CD8 + T cells, or tumor-associated macrophages as effectively as the triple combination, as confirmed by IHC staining.

Our findings suggest a potential synergy between RT, aPD-L1, and abemaciclib; however, the underlying mechanisms remain only partially understood. RT and PD-L1 blockade alone did not significantly enhance immune cell infiltration, likely due to persistent immunosuppressive factors within the tumor microenvironment. We propose that abemaciclib plays a critical role in overcoming this suppression by promoting T-cell activation and reprogramming macrophage populations toward a pro-inflammatory, immunostimulatory phenotype. Further investigations, including functional and mechanistic studies, are necessary to comprehensively elucidate these interactions.

While MCP-1, CD80, and CD206 were used as markers to characterize macrophage phenotypes, these markers have limitations. MCP-1, though associated with M1 macrophages, is context-dependent and linked to pro-tumorigenic roles such as recruiting monocytes that differentiate into M2-like macrophages [66]. CD80 and CD206, which are widely accepted markers for M1 and M2 macrophages, respectively [37, 38, 40], provide only a partial view of macrophage functionality. Therefore, we examined iNOS, a well-established functional hallmark of M1 macrophages, and found that its immunohistochemical staining pattern in both 4T1 and EMT6 tumors paralleled the enrichment of CD80 and MCP-1, further corroborating M1-skewed reprogramming in our triple combination (abemaciclib, RT, and aPD-L1)-treated mice [39, 67]. Nevertheless, the binary M1/M2 paradigm is increasingly regarded as an oversimplification because tumor-associated macrophages often display hybrid or transitional phenotypes along a continuum, relying on single markers risks misclassification. Current consensus guidelines therefore advocate multi-parameter validation, integrating additional markers (e.g., major histocompatibility complex class II, arginase 1) [67, 68], and functional read-outs together with transcriptomic or metabolic profiling to substantiate macrophage reprogramming.

A limitation of this study is that immune activation was evaluated only by serum IFN-γ. Chemokines in the CXCL9/10/11–CXCR3 axis, which are essential for recruiting effector CXCR3^+^ T cells and predicting the response to RT and ICI combinations, were not measured here [69, 70]. Likewise, tumor necrosis factor (TNF)-α can shape antigen presentation and T-cell function but exhibits context-dependent, bidirectional effects [71]. Future work will therefore include multiplex profiling of CXCL9/10/11, TNF-α, and related mediators to provide a more comprehensive view of treatment-induced immune modulation.

**Fig. 7:**
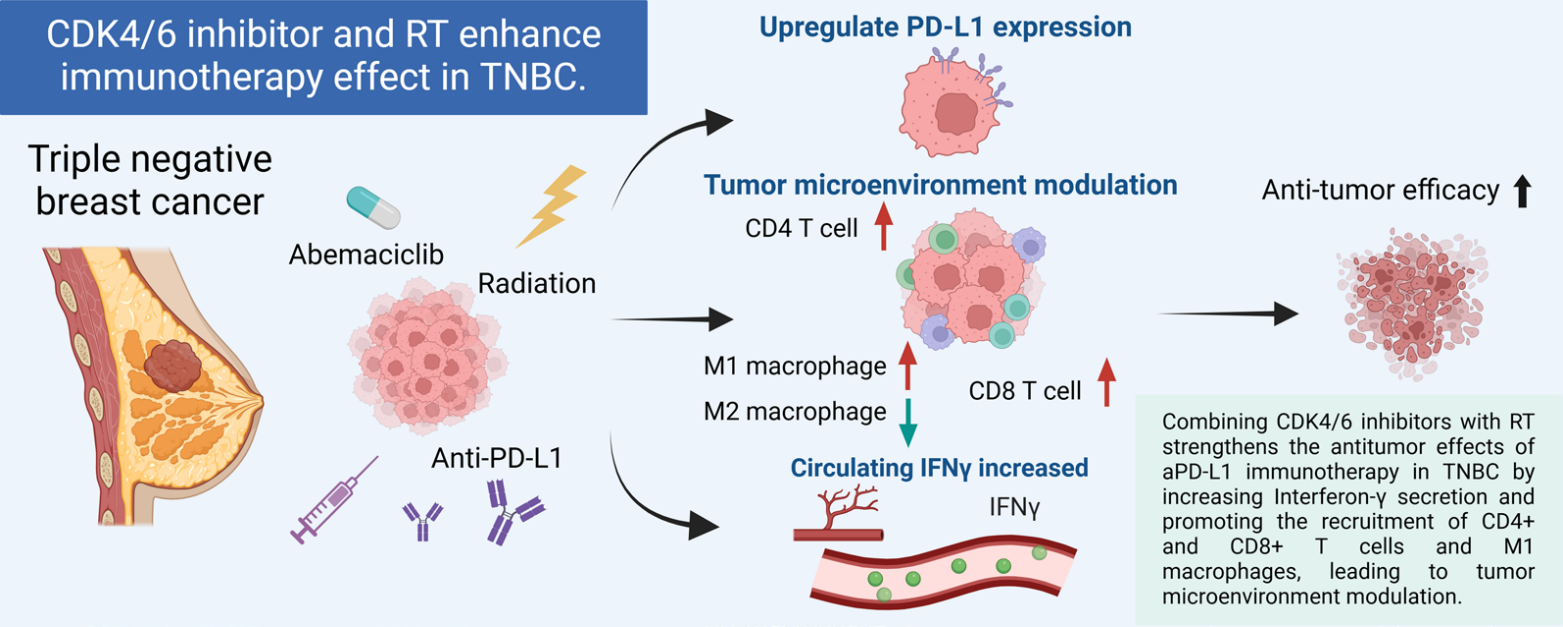
Modulation of the tumor microenvironment enhances antitumor efficacy in TNBC through triple combination therapy. The combination of CDK4/6 inhibitors, radiotherapy (RT), and anti-PD-L1 immunotherapy demonstrates potential antitumor effects in triple-negative breast cancer (TNBC). This treatment approach is associated with increased levels of circulating IFN-γ, enhanced recruitment of immune cells such as CD4 + and CD8 + T cells, and a shift in macrophage populations towards an immunostimulatory phenotype

The triple combination demonstrated a stronger correlation with increased immune cell infiltration, indicating a potential association with a more immunostimulatory tumor microenvironment (Fig. 7). This was reflected in the observed reduction in tumor size, wherein the triple combination showed the most substantial tumor growth inhibition among all treatment strategies in 4T1 and EMT6 murine TNBC cell line-derived xenograft immunocompetent mice.

## Conclusions

In the present study, we investigated the synergistic potential of combining CDK4/6 inhibitors, RT, and ICIs to improve the outcomes of TNBC treatment. Using 4T1 and EMT6 murine TNBC cell line-derived immunocompetent mouse models, our study demonstrated that the triple combination of abemaciclib, aPD-L1, and RT significantly reduced tumor growth. The triple combination treatment increased circulating IFN-γ levels and enhanced the recruitment of CD4 + T cells, CD8 + T cells, and M1 macrophages within the tumor microenvironment, potentially contributing to an improved antitumor immune response and tumor growth inhibition. Further exploration of the treatment efficacy of high-dose RT in combination with abemaciclib and ICIs in patients with advanced or metastatic TNBC is warranted.

## Ethic approval and consent to participate

The experimental procedures of animal models were approved by the Institutional Animal Care and Use Committee of the National Taiwan University College of Medicine and College of Public Health (IACUC Number 20210366).

## Consent for publication

Not applicable.

## Competing interests

The authors declare no competing interests.

## Supplementary Information

Additional file 1: Supplementary Fig. 1. The cell survival of MDA-MB-231, MDA-MB-453, and MDA-MB-468 was measured with Cell Proliferation Assay. IC50 values for abemaciclib were found to be 12.15, 2.86, and 8.01 μM for MDA-MB-231, MDA-MB-453, and MDA-MB-468 cells, respectively; and the corresponding cell survival curves were demonstrated. All experiments were performed three times.

Additional file 2: Supplementary Fig. 2. The expression of retinoblastoma (RB) and phosphorylated (p)-RB in five triple-negative breast cancer cell lines treated with different conditions of abemaciclib and radiotherapy (RT) using western blot analysis. All experiments were performed three times. The quantification data are presented with mean ± standard deviation. Significance levels: * *P* < 0.05; ** *P* < 0.01; *** *P* < 0.001; **** *P* < 0.0001.

Additional file 3: Supplementary Fig. 3. The gating strategy of tumor-infiltrating lymphocytes (TILs) in various treatments, including control, radiotherapy (RT), abemaciclib (Abe), anti-PD-L1 antibody (aPD-L1), Abe combined with aPD-L1, Abe combined with RT, aPD-L1 combined with RT, and the triple combination of Abe with aPD-L1 and RT. The M-MDSC gating strategy is listed separately.

Additional file 4: Supplementary Fig. 4. Immunohistochemical staining of PD-L1 in tumors (EMT6 immunocompetent mouse model) following treatment with abemaciclib, radiotherapy (RT), and anti-PD-L1 antibody (aPD-L1). Tumors excised from treated mice were stained for PD-L1. (A)-(H) display PD-L1 across different treatment groups, including the control, RT alone, abemaciclib alone, aPD-L1 alone, and their combinations. Scale bar: 50 μm. (I) shows the quantification of PD-L1 staining, presented as the average number of cells per high-power field. Statistical significance was assessed using an unpaired two-tailed t-test. Significance levels: *** *P* < 0.001; **** *P* < 0.0001.

Additional file 5: Supplementary Fig. 5. Immunohistochemical staining of CD4 + T cells, CD8 + T cells, MCP-1 + , CD80 + , iNOS + , and CD206 + macrophages in tumors (EMT6 immunocompetent mouse model) following treatment with abemaciclib, radiotherapy (RT), and anti-PD-L1 antibody (aPD-L1). Tumors excised from treated mice were stained for CD4, CD8, MCP-1, CD80, iNOS, and CD206. The upper panel displays representative images of (A) CD4 + , (B) CD8 + , (C) MCP-1 + , (D) CD80 + , (E) iNOS + , and (F) CD206 + cells across different treatment groups, including the control, RT alone, abemaciclib alone, aPD-L1 alone, and their combinations. Scale bar: 50 μm. The lower panel quantifies the average number of (G) CD4 + T cells, (H) CD8 + T cells, (I) MCP-1 + cells, (J) CD80 + , (K) iNOS + , and (L) CD206 + cells per high-power field. Statistical significance was assessed using an unpaired two-tailed t-test. Significance levels: * *P* < 0.05; ** *P* < 0.01; *** *P* < 0.001; **** *P* < 0.0001.

## Data Availability

No datasets were generated or analysed during the current study.

## Abbreviations

aPD-L1: Anti-programmed death-ligand 1 antibody
ATCC: American type culture collection
CDK4/6: Cyclin-dependent kinase 4 and 6
CI: Combination index
DMSO: Dimethyl sulfoxide
DMEM: Dulbecco’s Modified Eagle Medium
ELISA: Enzyme-linked immunosorbent assay
ER: Estrogen receptor
Foxp3: Forkhead box protein P3
GR-1: Granulocyte receptor-1
IC50: Half-maximal inhibitory concentration
HER2: Human epidermal growth factor receptor 2
ICI: Immune checkpoint inhibitor
IFN-γ: Interferon-gamma
IHC: Immunohistochemistry
IACUC: Institutional Animal Care and Use Committee
iNOS: Inducible nitric oxide synthase
MCP-1: Monocyte chemoattractant protein-1
MDSC: Myeloid-derived suppressor cell
M-MDSC: Monocytic myeloid-derived suppressor cell
PBS: Phosphate-buffered saline
PD-L1: Programmed death-ligand 1
PFS: Progression-free survival
p-RB: Phosphorylated retinoblastoma protein
RB: Retinoblastoma protein
RPMI: Roswell Park Memorial Institute medium
RT: Radiotherapy
SD: Standard deviation
TILs: Tumor-infiltrating lymphocytes
TNF-α: Tumor necrosis factor-α
TNBC: Triple-negative breast cance
Treg: Regulatory T cell
α-tubulin: Alpha tubulin
