# Supplementary material for: CDK4/6 inhibitors synergize with radiotherapy to prime the tumor microenvironment and enhance the antitumor effect of anti-PD-L1 immunotherapy in triple-negative breast cancer
Source: J Biomed Sci. 2025 Aug 20;32:79. doi: 10.1186/s12929-025-01173-3 (PMC12369063; doi:10.1186/s12929-025-01173-3)
Supplement: Supplementary file 1 — Additional file 1: Supplementary Fig. 1. The cell survival of MDA-MB-231, MDA-MB-453, and MDA-MB-468 was measured with Cell Proliferation Assay. IC50 values for abemaciclib were found to be 12.15, 2.86, and 8.01 μM for MDA-MB-231, MDA-MB-453, and MDA-MB-468 cells, respectively; and the corresponding cell survival curves were demonstrated. All experiments were performed three times. [file 12929_2025_1173_MOESM1_ESM.docx]

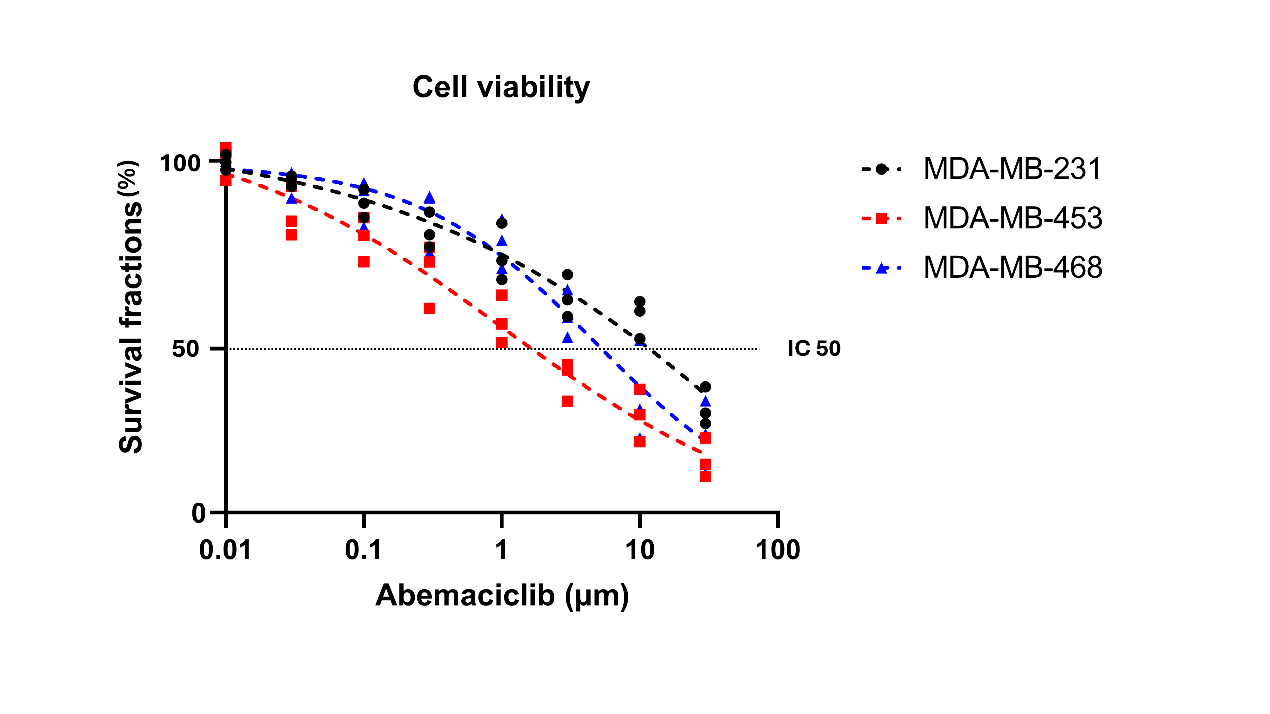


**Supplementary Figure 1.** The cell survival of MDA-MB-231, MDA-MB-453, and MDA-MB-468 was measured with Cell Proliferation Assay. IC50 values for abemaciclib were found to be 12.15, 2.86, and 8.01 μM for MDA-MB-231, MDA-MB-453, and MDA-MB-468 cells, respectively; and the corresponding cell survival curves were demonstrated. All experiments were performed three times.
