# Supplementary material for: CDK4/6 inhibitors synergize with radiotherapy to prime the tumor microenvironment and enhance the antitumor effect of anti-PD-L1 immunotherapy in triple-negative breast cancer
Source: J Biomed Sci. 2025 Aug 20;32:79. doi: 10.1186/s12929-025-01173-3 (PMC12369063; doi:10.1186/s12929-025-01173-3)
Supplement: Supplementary file 2 — Additional file 2: Supplementary Fig. 2. The expression of retinoblastoma (RB) and phosphorylated (p)-RB in five triple-negative breast cancer cell lines treated with different conditions of abemaciclib and radiotherapy (RT) using western blot analysis. All experiments were performed three times. The quantification data are presented with mean ± standard deviation. Significance levels: * P < 0.05; ** P < 0.01; *** P < 0.001; **** P < 0.0001. [file 12929_2025_1173_MOESM2_ESM.docx]

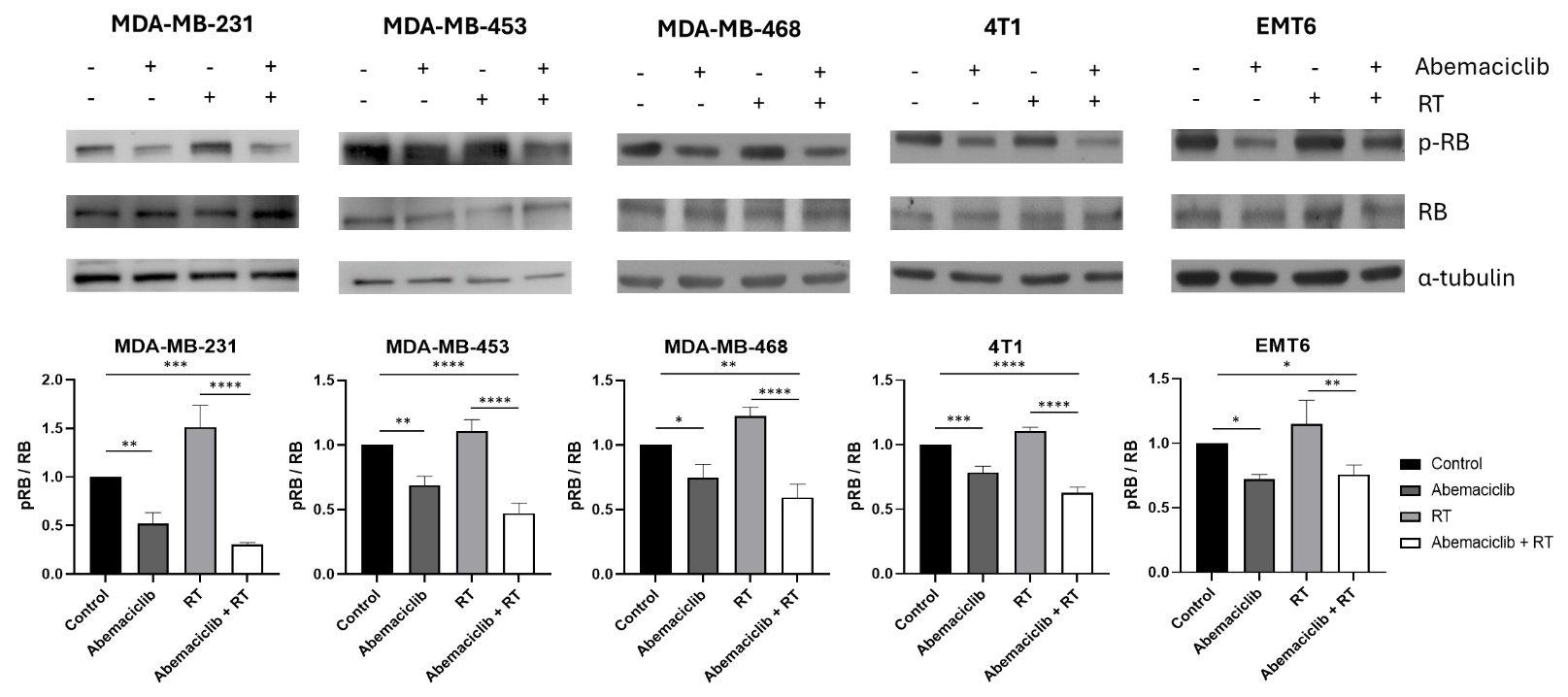


**Supplementary Figure 2.** The expression of retinoblastoma (RB) and phosphorylated (p)-RB in five triple-negative breast cancer cell lines treated with different conditions of abemaciclib and radiotherapy (RT) using western blot analysis. All experiments were performed three times. The quantification data are presented with mean ± standard deviation. Significance levels: * *P <* 0.05; ** *P <* 0.01; *** *P <* 0.001; **** *P <* 0.0001.
