# Supplementary material for: CDK4/6 inhibitors synergize with radiotherapy to prime the tumor microenvironment and enhance the antitumor effect of anti-PD-L1 immunotherapy in triple-negative breast cancer
Source: J Biomed Sci. 2025 Aug 20;32:79. doi: 10.1186/s12929-025-01173-3 (PMC12369063; doi:10.1186/s12929-025-01173-3)
Supplement: Supplementary file 3 — Additional file 3: Supplementary Fig. 3. The gating strategy of tumor-infiltrating lymphocytes (TILs) in various treatments, including control, radiotherapy (RT), abemaciclib (Abe), anti-PD-L1 antibody (aPD-L1), Abe combined with aPD-L1, Abe combined with RT, aPD-L1 combined with RT, and the triple combination of Abe with aPD-L1 and RT. The M-MDSC gating strategy is listed separately. [file 12929_2025_1173_MOESM3_ESM.docx]

**Supplementary Figure 3.** The gating strategy of tumor-infiltrating lymphocytes (TILs) in various treatments, including control, radiotherapy (RT), abemaciclib (Abe), anti-PD-L1 antibody (aPD-L1), Abe combined with aPD-L1, Abe combined with RT, aPD-L1 combined with RT, and the triple combination of Abe with aPD-L1 and RT. The monocytic*-*myeloid-derived suppressor cell (M-MDSC) gating strategy is listed separately.


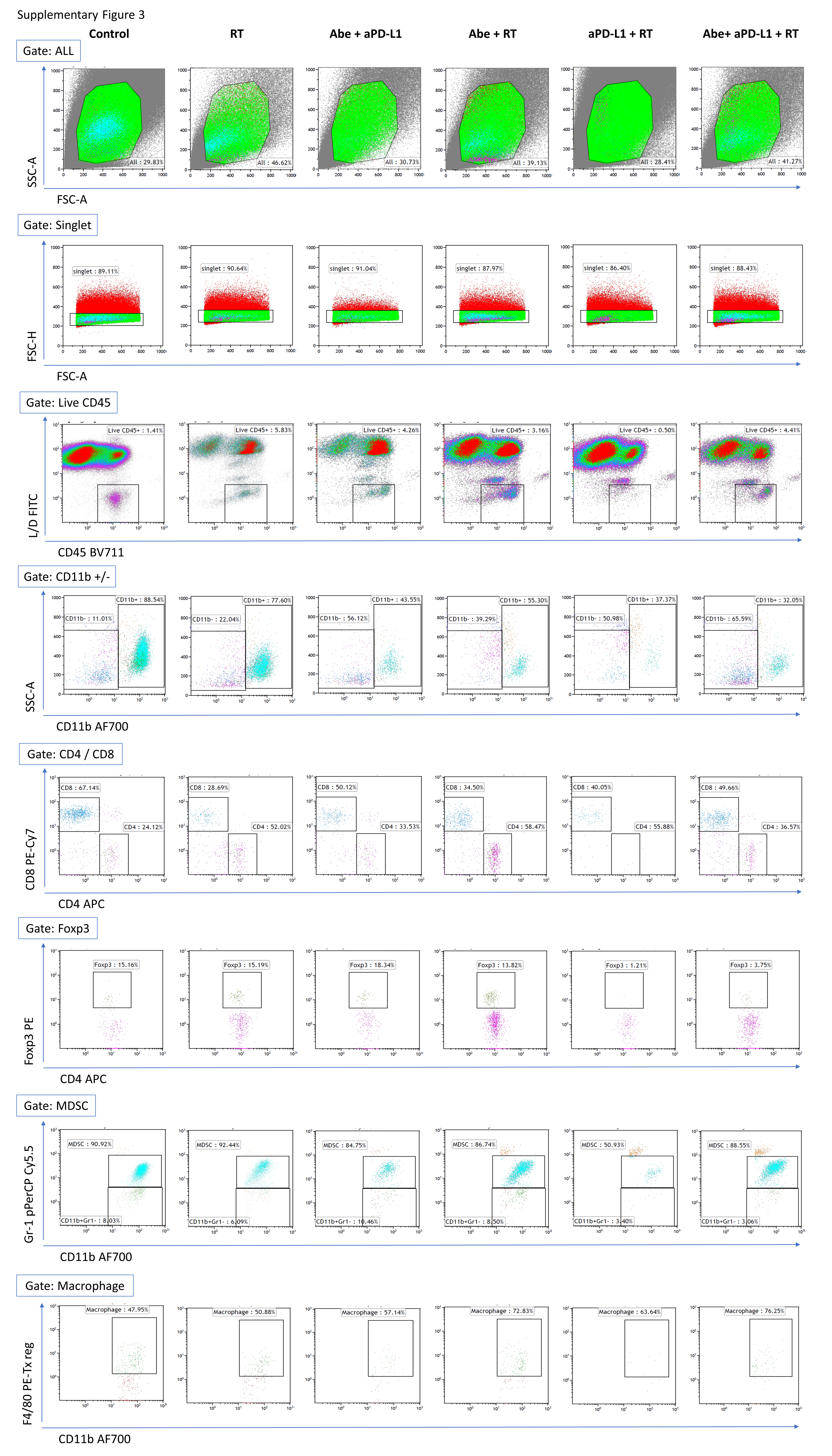


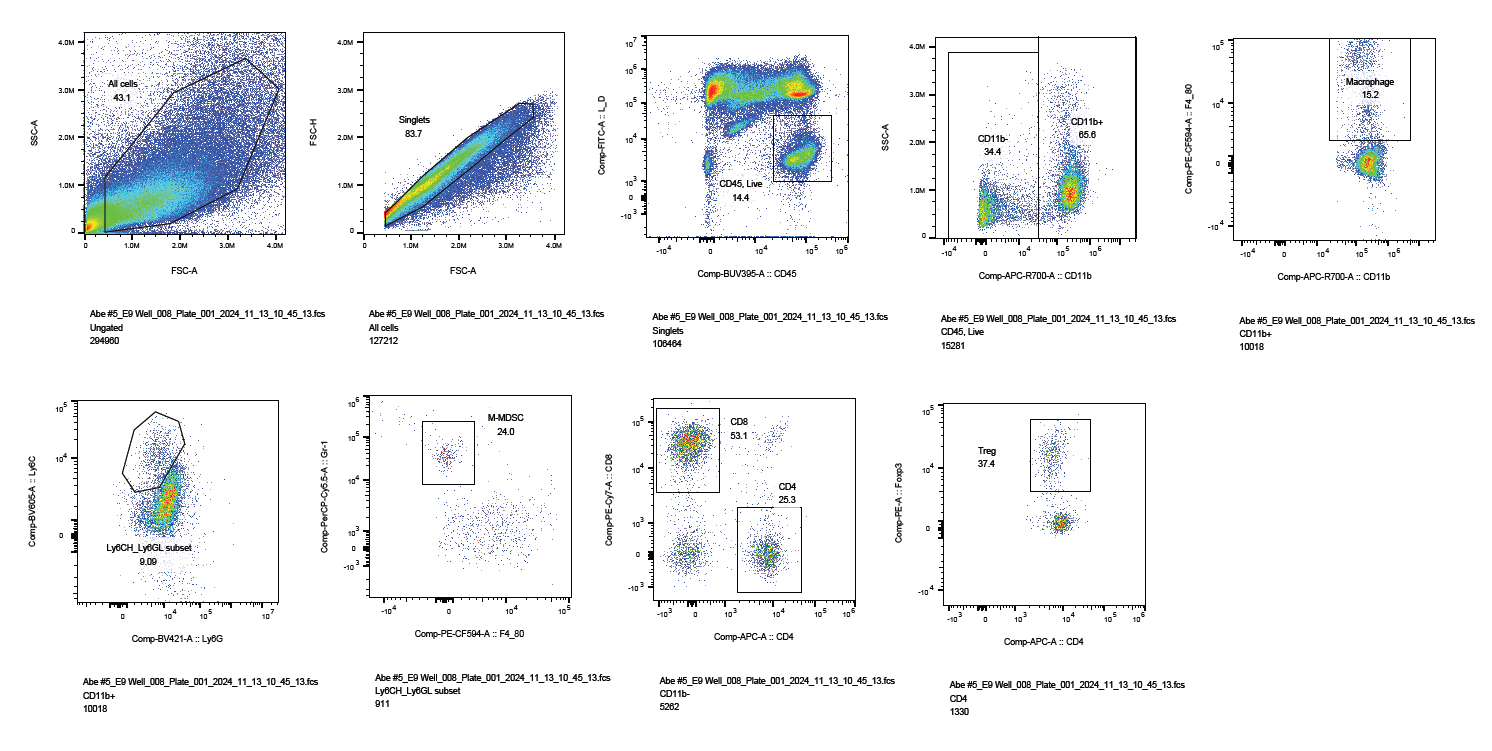
**Gating: Abemaciclib group**


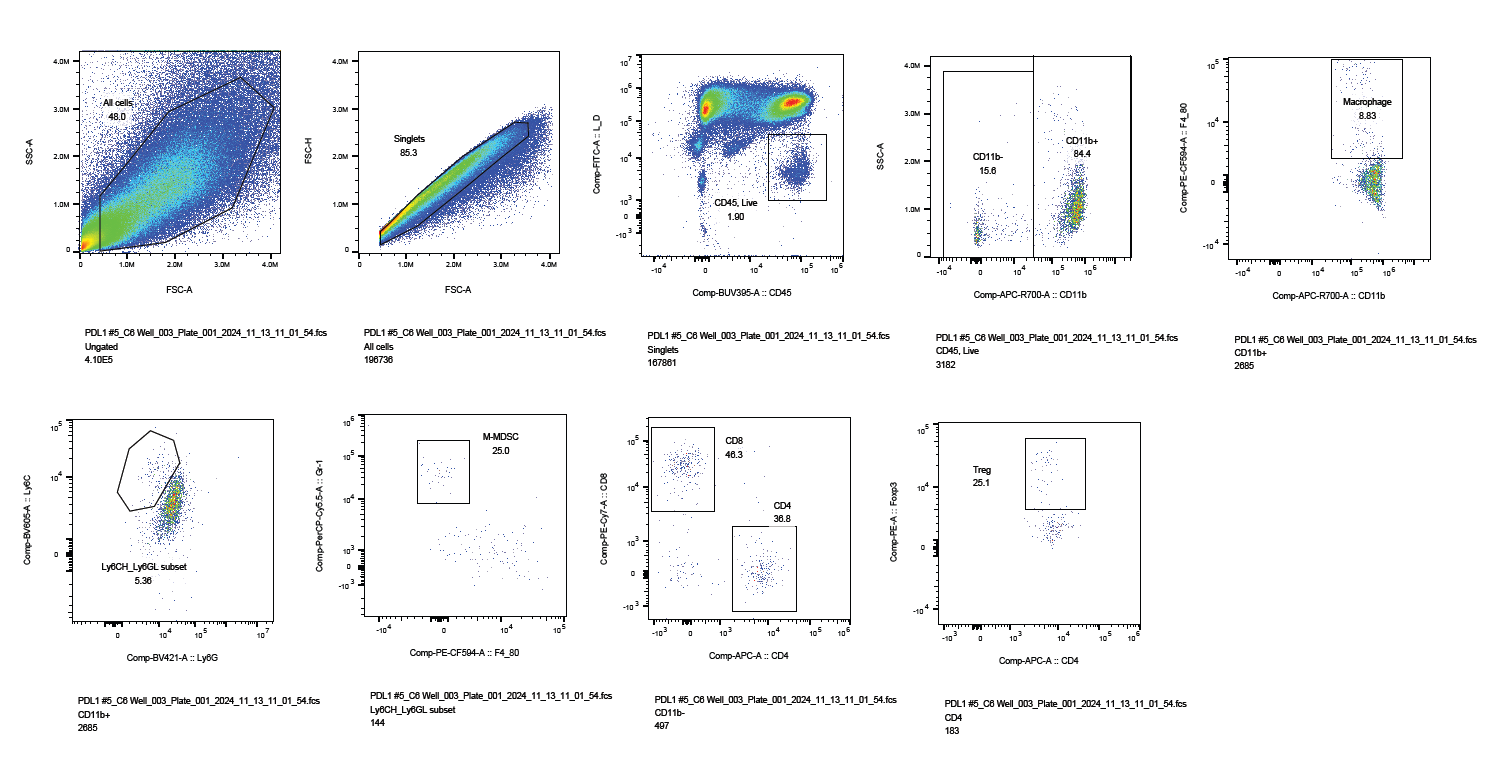
**Gating: aPD-L1 group**

**M-MDSC gating for other groups (control, radiotherapy [RT], abemaciclib [Abe] + aPD-L1, Abe+ RT, aPD-L1+ RT, Abe+ aPD-L1 +RT)**

**
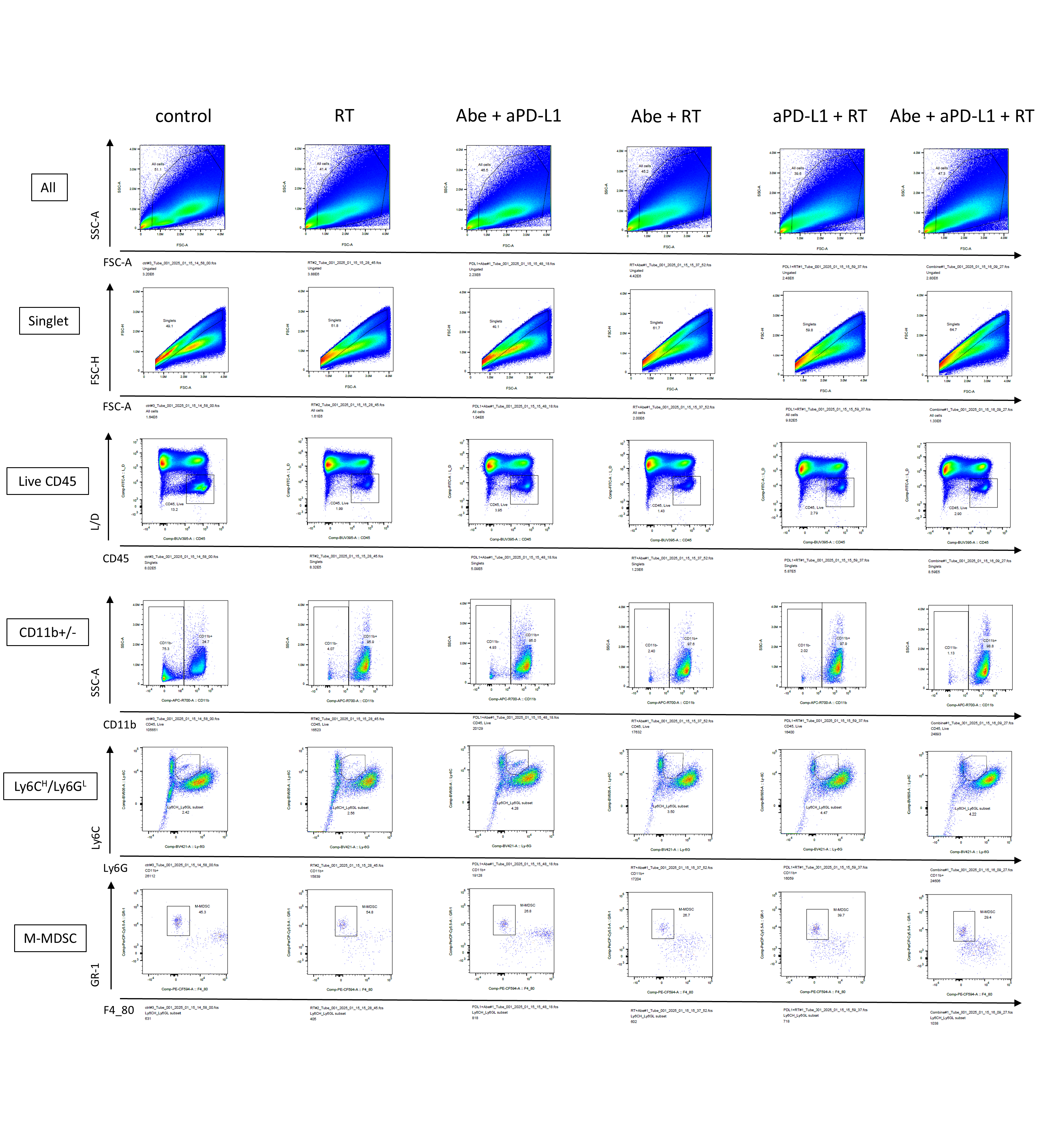
**
