# Supplementary material for: CDK4/6 inhibitors synergize with radiotherapy to prime the tumor microenvironment and enhance the antitumor effect of anti-PD-L1 immunotherapy in triple-negative breast cancer
Source: J Biomed Sci. 2025 Aug 20;32:79. doi: 10.1186/s12929-025-01173-3 (PMC12369063; doi:10.1186/s12929-025-01173-3)
Supplement: Supplementary file 4 — Additional file 4: Supplementary Fig. 4. Immunohistochemical staining of PD-L1 in tumors (EMT6 immunocompetent mouse model) following treatment with abemaciclib, radiotherapy (RT), and anti-PD-L1 antibody (aPD-L1). Tumors excised from treated mice were stained for PD-L1. (A)-(H) display PD-L1 across different treatment groups, including the control, RT alone, abemaciclib alone, aPD-L1 alone, and their combinations. Scale bar: 50 μm. (I) shows the quantification of PD-L1 staining, presented as the average number of cells per high-power field. Statistical significance was assessed using an unpaired two-tailed t-test. Significance levels: *** P < 0.001; **** P < 0.0001. [file 12929_2025_1173_MOESM4_ESM.docx]

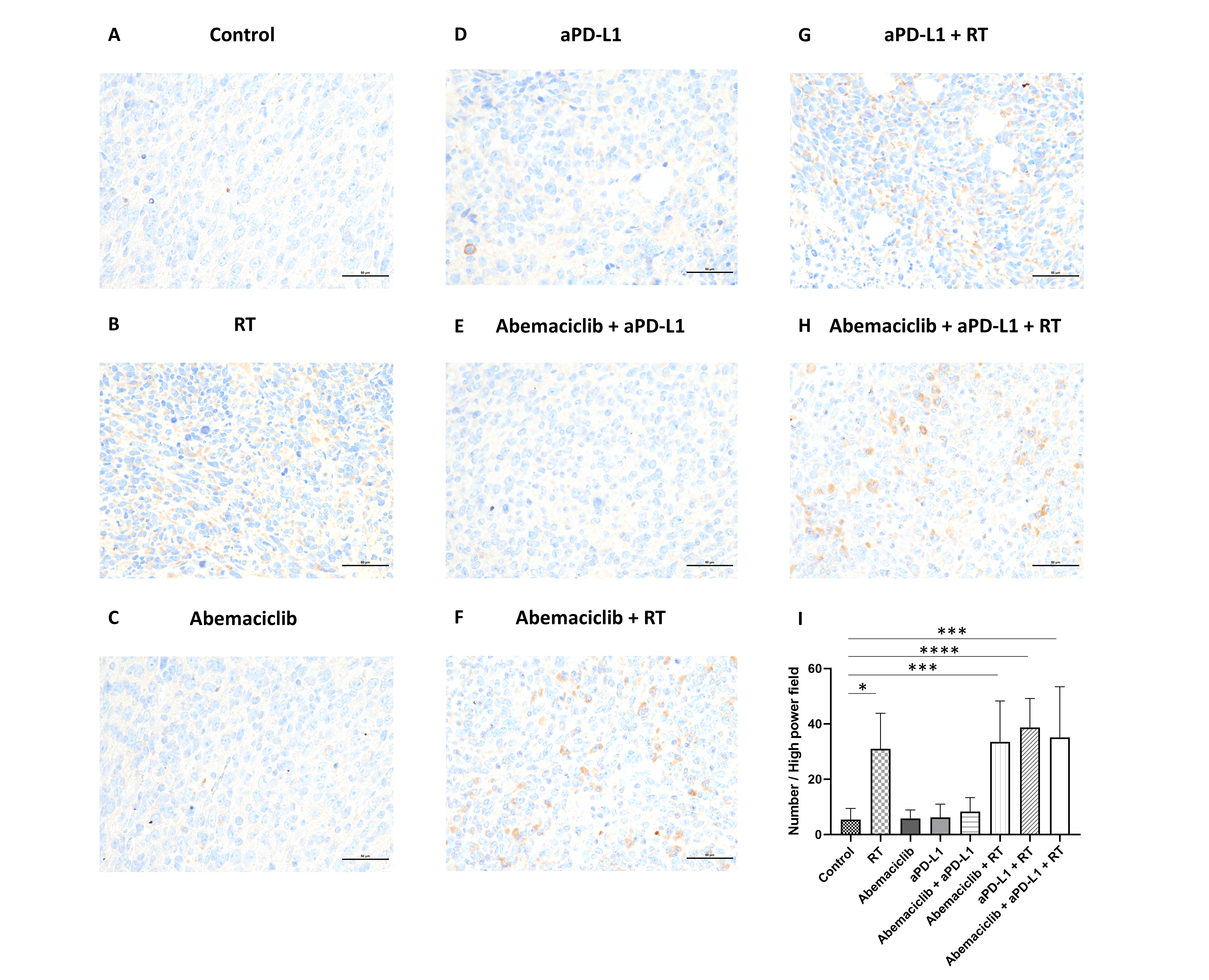


**Supplementary Figure 4. Immunohistochemical staining of PD-L1 in tumors (EMT6 immunocompetent mouse model) following treatment with abemaciclib, radiotherapy (RT), and anti-PD-L1 antibody (aPD-L1).** Tumors excised from treated mice were stained for PD-L1. (A)-(H) display PD-L1 across different treatment groups, including the control, RT alone, abemaciclib alone, aPD-L1 alone, and their combinations. Scale bar: 50 μm. (I) shows the quantification of PD-L1 staining, presented as the average number of cells per high-power field. Statistical significance was assessed using an unpaired two-tailed t-test. Significance levels: *** *P <* 0.001; **** *P <* 0.0001.
