# Supplementary material for: CDK4/6 inhibitors synergize with radiotherapy to prime the tumor microenvironment and enhance the antitumor effect of anti-PD-L1 immunotherapy in triple-negative breast cancer
Source: J Biomed Sci. 2025 Aug 20;32:79. doi: 10.1186/s12929-025-01173-3 (PMC12369063; doi:10.1186/s12929-025-01173-3)
Supplement: Supplementary file 5 — Additional file 5: Supplementary Fig. 5. Immunohistochemical staining of CD4 + T cells, CD8 + T cells, MCP-1 + , CD80 + , iNOS + , and CD206 + macrophages in tumors (EMT6 immunocompetent mouse model) following treatment with abemaciclib, radiotherapy (RT), and anti-PD-L1 antibody (aPD-L1). Tumors excised from treated mice were stained for CD4, CD8, MCP-1, CD80, iNOS, and CD206. The upper panel displays representative images of (A) CD4 + , (B) CD8 + , (C) MCP-1 + , (D) CD80 + , (E) iNOS + , and (F) CD206 + cells across different treatment groups, including the control, RT alone, abemaciclib alone, aPD-L1 alone, and their combinations. Scale bar: 50 μm. The lower panel quantifies the average number of (G) CD4 + T cells, (H) CD8 + T cells, (I) MCP-1 + cells, (J) CD80 + , (K) iNOS + , and (L) CD206 + cells per high-power field. Statistical significance was assessed using an unpaired two-tailed t-test. Significance levels: * P < 0.05; ** P < 0.01; *** P < 0.001; **** P < 0.0001. [file 12929_2025_1173_MOESM5_ESM.docx]

**Supplementary Figure 5. Immunohistochemical staining of CD4+ T cells, CD8+ T cells, MCP-1-, CD80-, iNOS-, and CD206-positive macrophages in tumors (****EMT6 immunocompetent mouse model) following treatment with abemaciclib, radiotherapy (RT), and anti-PD-L1 antibody (aPD-L1).** Tumors excised from treated mice were stained for CD4, CD8, MCP-1, CD80, iNOS, and CD206. The upper panel displays representative images of (A) CD4+, (B) CD8+, (C) MCP-1+, (D) CD80+, (E) iNOS+, and (F) CD206+ cells across different treatment groups, including the control, RT alone, abemaciclib alone, aPD-L1 alone, and their combinations. Scale bar: 50 μm. The lower panel quantifies the average number of (G) CD4+ T cells, (H) CD8+ T cells, (I) MCP-1+ cells, (J) CD80+, (K) iNOS+, and (L) CD206+ cells per high-power field. Statistical significance was assessed using an unpaired two-tailed t-test. Significance levels: ** *P <* 0.01; *** *P <* 0.001; **** *P <* 0.0001.
